# *Staphylococcus aureus* induces tolerance in human monocytes accompanied with expression changes of cell surface markers

**DOI:** 10.3389/fimmu.2023.1046374

**Published:** 2023-03-31

**Authors:** Mario M. Müller, Christian Baldauf, Stella Hornischer, Tilman E. Klassert, Antony Schneegans, Andrea Behnert, Mathias W. Pletz, Stefan Hagel, Hortense Slevogt

**Affiliations:** ^1^Septomics Research Center, Jena University Hospital, Jena, Germany; ^2^Integrated Research and Treatment Center - Center for Sepsis Control and Care (CSCC), Jena University Hospital, Jena, Germany; ^3^Department of Respiratory Medicine, Hannover Medical School, Hannover, Germany; ^4^Institute for Infectious Diseases and Infection Control, Jena University Hospital – Friedrich Schiller University Jena, Jena, Germany

**Keywords:** proteomics, glycoproteins, *S. aureus*, tolerance, monocyte, membrane proteins, marker of tolerance, bacteremia

## Abstract

Exposure of human monocytes to lipopolysaccharide (LPS) or other pathogen-associated molecular pattern (PAMPs) induces a temporary insensitivity to subsequent LPS challenges, a cellular state called endotoxin tolerance (ET), associated with the pathogenesis of sepsis. In this study, we aimed to characterize the cellular state of human monocytes from healthy donors stimulated with *Staphylococcus aureus* in comparison to TLR2-specific ligands. We analyzed *S. aureus* induced gene expression changes after 2 and 24 hours by amplicon sequencing (RNA-AmpliSeq) and compared the pro-inflammatory response after 2 hours with the response in re-stimulation experiments. In parallel, glycoprotein expression changes in human monocytes after 24 hours of *S. aureus* stimulation were analyzed by proteomics and compared to stimulation experiments with TLR2 ligands Malp-2 and Pam3Cys and TLR4 ligand LPS. Finally, we analyzed peripheral blood monocytes of patients with *S. aureus* bloodstream infection for their *ex vivo* inflammatory responses towards *S. aureus* stimulation and their glycoprotein expression profiles. Our results demonstrate that monocytes from healthy donors stimulated with *S. aureus* and TLR ligands of Gram-positive bacteria entered the tolerant cell state after activation similar to LPS treatment. In particular reduced gene expression of pro-inflammatory cytokines (TNF, IL1β) and chemokines (CCL20, CCL3, CCL4, CXCL2, CXCL3 and CXCL8) could be demonstrated. Glycoprotein expression changes in monocytes tolerized by the different TLR agonists were highly similar while *S. aureus*-stimulated monocytes shared some of the PAMP-induced changes but also exhibited a distinct expression profile. 11 glycoproteins (CD44, CD274, DSC2, ICAM1, LAMP3, LILRB1, PTGS2, SLC1A3, CR1, FGL2, and HP) were similarly up- or downregulated in all four comparisons in the tolerant cell state. Monocytes from patients with *S. aureus* bacteremia revealed preserved pro-inflammatory responsiveness to *S. aureus* stimulation ex vivo, expressed increased CD44 mRNA but no other glycoprotein of the tolerance signature was differentially expressed.

## Introduction

Monocytes and macrophages play a major role in the pathogenesis of Gram-positive and Gram-negative bacteria induced systemic infections. In the blood stream, invading bacteria are cleared by phagocytic cells and are recognized *via* Toll-like receptors (TLRs) expressed by innate immune cells interacting with pathogen-associated molecular patterns (PAMPs) (1–3). The initial recognition of PAMPs triggers pro-inflammatory signaling in monocytes and macrophages to elicit an adequate immune response. Lipopolysaccharides (LPS), which constitutes one major cell wall component of Gram-negative bacteria, interacts with the TLR4 receptor complex to induce intracellular Nuclear-factor kappa B (NFκB) activation and subsequent transcriptional activation of pro-inflammatory cytokines. Long-term exposure to LPS or injection of sub lethal doses into animals has been shown to induce a state of immunosuppression, called endotoxin tolerance (ET) that reprograms the inflammatory responses of TLR-mediated activation, resulting in reduced pro-inflammatory cytokine production in subsequent challenges with LPS or other inflammatory stimuli (4, 5). Due to epigenetic reprograming mechanisms ET is characterized by substantial changes in gene transcription, with extenuated expression of pro-inflammatory cytokines and chemokines, often termed ‘tolerizable genes’ (T-genes), and increased expression of a different subset of genes (‘non-tolerizable genes’, NT-genes) that may protect the host from an overwhelming immune response during ongoing systemic infections (4, 6–9). Endotoxin-tolerant monocytes exhibit increased phagocytic capability, but impaired antigen-presentation and chemotaxis capacity, while maintaining their killing competence of internalized pathogens (10, 11). Thus, ET establishes a state of immune memory that is adaptive against systemic infections protecting the host from exuberant inflammation (12).

Similar to LPS, cell wall components from Gram-positive bacteria, e.g. di- and triacyl lipopeptides, lipoteichoic acids, macrophage-activating peptide-2 (MALP2) or intact Gram-positive bacteria are also capable to induce the tolerant cell state in monocytes *via* the TLR1/TLR2 or TLR2/TLR6 signaling pathways and epigenetic alterations (13–18) with protective effects during sepsis in mouse models (15, 19). *S. aureus* is a Gram-positive pathogen that can cause superficial skin infections, serious invasive infections such as septic arthritis, osteomyelitis or endocarditis and is one of the leading causes of life-threatening bloodstream infections, such as sepsis (20, 21). It is ranked second after *Escherichia coli* causing community-acquired and nosocomial bloodstream infections with 10 to 30 cases per 100.000 person-years (22, 23). The associated fatality rate of 10-30% leads to a calculated mortality of 2-10 deaths per 100.000 individuals in the population (24, 25). The Gram-positive pathogen can infect virtually any tissue or organ and readily growth on artificial surfaces of implanted foreign bodies or intravascular or urinary catheters. Infections with *S. aureus* can become chronic or may relapse, despite appropriate antibiotic treatment. For instance, in up to 16% of all cases with reported *S. aureus* bacteremia relapses were described, and in one-third of all patients *S. aureus* can persist in blood for several days even with high-dosage intravenous antibiotic therapy (26, 27). The pathogen has developed several strategies to escape immune recognition and clearance, e.g. by forming biofilms, or adapting to intracellular growth conditions within host cells by changing metabolism and morphology towards slow growing small colony variants which show also increased tolerance against antibiotics and can result in refractory or chronic infections (28, 29). Whole *S. aureus* cell wall extracts as well as specific *S. aureus*-derived cell membrane compounds like peptidoglycan or glycan-teichoic acid have profound effects on monocyte activation with regard to pro-inflammatory cytokine production and secretion (13), as well as TLR2-mediated tolerance induction (18).

Tolerant monocytes with diminished capacity to release pro-inflammatory cytokines have been observed in patients with sepsis and septic shock and this cell state is the most prominent feature of sepsis-related immunosuppression (7, 30). Magnitude and persistence of this refractory state are associated with increased mortality and occurrence of nosocomial infections (30, 31). Differences in monocyte cell surface marker expression have been suggested as surrogate markers for the immunosuppressive tolerant state and reduced expression of major histocompatibility complex molecule II (MHC) HLA-Dr, or upregulated expression of Programmed cell death 1 ligand 1 (PD1L1) are well documented and already discussed as markers for individualized sepsis therapy (32–35). We and others have recently shown that global analysis of enriched glycoproteins by proteomics and mass spectrometry is a suitable method for comparing cellular receptor expression profiles (36–38), and recently, we reported a cell surface signature of human monocytes tolerized by LPS using a global glycoproteomic approach (37). Less is known about the potential of vital *S. aureus* to induce tolerance in human monocytes and possibly associated receptor expression changes.

In this study, we analyzed the potential of tolerance induction by *S. aureus* stimulation in comparison to TLR2- or TLR4-specific ligands in human monocytes from healthy donors. Transcriptional changes in the pro-inflammatory and the presumed refractory cell state were analyzed by amplicon sequencing with an in-house build primer library. Monocyte glycoprotein expression profiles after *S. aureus* or TLR2 agonist stimulation were compared to unstimulated monocytes and among each other after glycoprotein purification and LC-MS/MS analysis (37, 39). We found that *S. aureus* stimulation induced a tolerant cell state accompanied with extensive transcriptional alterations in human monocytes and we report a glycoprotein signature differentially expressed in the tolerant cell state after TLR2 agonist or *S. aureus* stimulation. Lastly, we found no indication for the development of the tolerant cell state in monocytes from patients with *S. aureus* bacteremia in *ex vivo* stimulation experiments. Patient-derived monocytes expressed increased mRNA levels of the activation marker CD44, while signature glycoproteins of tolerance or other activation markers were not detected.

## Materials and methods

### Blood samples and patients

Human peripheral monocytes were purified from freshly drawn blood samples (50 ml) from ten healthy adult volunteers (*S. aureus* experiments), or three human buffy coats (TLR agonist stimulation experiments), or from patients with proven *S. aureus* bacteremia (Jena University Hospital). Patients were included if the following criteria were fulfilled: age ≥18, *S. aureus* bacteremia, signed consent to the study. Exclusion criteria were: pregnancy, high dosage corticoid therapy, chemotherapy, malignant tumors, HIV or AIDS. 2-4 days after the first *S. aureus* positive blood culture, blood samples (5-10 ml) were obtained and immediately transferred to the laboratory for further analysis. The study protocol was approved by the ethics committee of the Friedrich Schiller University Jena (4690-02/16 and 5189-06/17). Patient characteristics can be found in **Supplementary Table S1**.

### Isolation of monocytes

Peripheral blood monocytes were purified from EDTA-vacutainer blood collection tubes or buffy coats. In brief, PBMCs were enriched with Leucosep separation tubes and Ficoll gradient medium. The PBMC-containing fraction was washed and erythrocytes removed with ACK lysis buffer. The resulting cell suspension was used for MACS-bead-based negative isolation of monocytes (Monocyte isolation Kit II, Miltenyi Biotech) according to the manufacturer’s instructions with the exception of 2 mM EDTA supplementation in all buffers. MACS purified monocytes were used for all experiments except for glycoproteomic studies. For glycoproteomic analysis monocytes were further purified by flow cytometry-assisted cell sorting. Classical and non-classical monocytes were labeled with anti-CD14-FITC and anti-CD16-APC antibodies (eBioscience) and contaminating T-cells, B-cells, Granulocytes, Platelets and NK-cells were labeled with anti-CD3, anti-CD19 (both BD Biosciences), anti-CD66b (Miltenyi Biotec), anti-CD42b and anti-CD56 antibodies (both eBioscience) all conjugated with PE. PE positive particles and CD16-positive non-classical monocytes were excluded and CD14^+^ CD16^-^ monocytes were sorted into PBS using a BD FACSAria II (BD Biosciences, Germany). Monocytes were cultured in RPMI medium (Invitrogen, UK) supplemented with 10% fetal calf serum (Invitrogen, UK) and Penicillin and Streptomycin. For tolerance induction and qPCR analysis of healthy donor monocytes, 2 x 10^6^ cells were either left untreated or pre-stimulated with 50 ng/ml LPS, 100 ng/ml Pam3Cys, 10 ng/ml Malp-2 or *S. aureus* 6850 (MOI = 5). Cells were collected by centrifugation and resuspended in fresh medium with or without LPS or *S. aureus* 6850 (MOI=5) for restimulation. The second stimulation was allowed to proceed for 2 hours and, subsequently, cells were lysed in RLT buffer (Qiagen, Germany). Purified monocytes from patients were divided in two parts for RNA-AmpliSeq and glycoproteomic experiments. For amplicon sequencing half of the sample was cultured for 2h without any treatment, the other was stimulated with S. aureus (MOI=5) for 2h before lysis. For glycoproteomics patient monocytes were sorted by flow cytometry and directly lysed in 2%SDS/PBS. For ELISA testing 100.000 monocytes were seeded in a 96well plate and stimulated for 24h. Cells were centrifuged and culture supernatant was collected. For restimulation experiments, cells were resuspended in fresh medium, washed, and replated. Restimulation with *S. aureus* or LPS was done for another 24h period. TNF and IL6 were detected using ELISA kits Human TNF ELISA Set (BD Biosciences) and Human IL-6 ELISA (eBiosciences) according to the manufacturer’s instructions.

### Quantitative RT-PCR

Total RNA was isolated from 2 × 10^6^ cells using the InnuPREP RNA mini kit from Analytik Jena (Jena, Germany). Residual genomic DNA was degraded by DNaseI (Qiagen, Germany). RNA concentration was measured with a NanoDrop D-1000 Spectrophotometer (Thermo-Fisher Scientific, Germany). Complementary DNA (cDNA) was synthesized from 2 μg RNA using the High Capacity cDNA Reverse Transcription Kit (Applied Biosystems, UK) following the manufacturer’s instructions. The sequences of primers used for amplification were: TNFa, fw: TTCTCCTTCCTGATCGTGGC, rv: ACTCGGGGTTCGAGAAGATG; IL6, fw: GAGGAGACTTGCCTGGTGAA, rv: TGGGTCAGGGGTGGTTATTG; LILRB1, fw: CTGTTACTATGGTAGCGACACTG, rv: CTGAGAGGGTGGGTTTGATGT; CCRL2, fw: TTGGACTGTACTTCGTGGGC, rv: TGTTACCCATGCCAGGACAC; tmCD44, fw: TGGCTGATCATCTTGGCATCC, rv: CCGTTGAGTCCACTTGGCTT; CCL20, fw GTCTGTGTGCGCAAATCCAA, rv: GCAAGTGAAACCTCCAACCC; IL1B, fw: AGGAAGATGCTGGTTCCCTG, rv: GCATCGTGCACATAAGCCTC. To quantify the relative expression of each gene, a Corbett Rotor-Gene 6000 (Qiagen, Germany) was used for real-time qPCR. Each sample was analyzed in duplicate in a total reaction volume of 20 μl containing 10 μl of 2 × SensiMix SYBR Master Mix (Bioline, UK) and 0.2 μM of each primer. All qPCRs reactions were assembled using the CAS-1200 pipetting robot (Qiagen, Germany). The cycling conditions were 95°C for 10 min followed by 40 cycles of 95°C for 15 s, 60°C for 20 s and 72°C for 20 s. For each experiment, an RT-negative sample was included as control. Specificity of the qPCRs was assessed by melting curve analysis. Relative expression of target genes was analyzed using a modified method described by Pfaffl et al. (40). The stability of the housekeeping genes PPIB and HPRT was assessed using the BestKeeper algorithm (41).The normalized RQ (NRQ) values were log2-transformed for further statistical analysis with GraphPad PRISM v5.0 software. Comparative analysis between groups was performed by 1-way ANOVA using the Bonferroni method for multiple test correction.

### RNA AmpliSeq

Total RNA from monocytes was quantified using a Nanodrop ND-1000 spectrophotometer.

RNA Ampliseq libraries were prepared using the Ion AmpliSeq™ Library Kits 2.0 (Thermo-Fisher Scientific, Germany) and a custom primer panel addressing 936 target genes involved in monocyte/macrophage immune functions and tolerance (based on references (11, 37, 42, 43), plus housekeeping genes and in-house specific targets, see also **Supplementary Table S2**, designed and produced by ThermoFisher). In short, 100 ng cDNA of reverse-transcribed was used for the initial multiplex PCR using a high fidelity polymerase mix and the following thermal cycling conditions: denaturation at 95°C, annealing at 60°C, elongation at 72°C (15 cycles). After the PCR reaction FuPa was added to partially digest primer sequences and phosphorylate amplicons. Next, Ion P1 adaptors and Ion Express barcodes were ligated to the amplicons. Barcoded amplicons were purified using Agencourt AMPure XP beads (Ambion), and quantified by qPCR. Individual libraries were then pooled equimolarly and the final library concentration adjusted to 100 pM of DNA. The pooled library templates were then clonally amplified on Sphere particles using an Ion Chef instrument (Thermo-Fisher Scientific) with Ion PI Hi-Q sequencing chemistry. Finally, they were loaded on Ion PI chips and sequenced on an Ion Proton apparatus (Thermo Fisher Scientific). The raw sequencing reads in fastq format were quality-checked using FastQC. Mapping of the reads was performed with the TMAP aligner, which was specifically developed to map IonTorrent data. Bed-files were prepared to cover the exact gene regions targeted by the primer panel. The resulting BAM-files were used as input for the Partek Genomics Suite (v. 6.6) to generate quantitative data (total counts and RPKM values). We set the lower detection limit to an average of five reads per amplicon in order to reduce artefacts in the lower detection range. RPKM values of each gene were then normalized against the geometric mean of four house-keeping genes (TBP, PIGG, HPRT1 and PPIB) included in the AmpliSeq panel.

### Glycoprotein enrichment for glycoproteomics

Samples for glycoprotein analysis were prepared from three different donors (PAMP stimulation experiments), or seven different donors (*S. aureus* stimulation experiments), and 10 different patients. Glycoprotein enrichment was done as described previously (37). In brief, 100 µl of monocyte lysate was treated with Benzonase nuclease (0.5 µl, Sigma-Aldrich) for 1 h at 37°C and centrifuged at 24.000 x g for 20 min (20°C) to pellet insoluble material. The supernatant was subjected to buffer exchange using polyacrylamide spin desalting columns (7K MWCO, Pierce) equilibrated with 100 mM sodium acetate, pH 5.5. Samples were oxidized with 10 mM sodium (meta)periodate (Sigma-Aldrich) for 30 min at room temperature in the dark. Sodium periodate was removed by buffer exchange using polyacrylamide desalting spin columns equilibrated with PBS pH=7.5. Oxidized sugar groups were immobilized on 50 µl (bead volume) Ultralink Hydrazide Gel (Pierce) overnight at room temperature with aniline (Sigma-Aldrich) as catalyst. Next day, non-glycoproteins were removed by washing three times with each of the following buffers: 1% SDS/PBS, 8M Urea in 100 mM Tris/HCl pH=8.0, 1 M NaCl in 100 mM Tris/HCl pH=8.0, 20% CH_3_CN/10 mM Tris/HCl pH=8.0. After the second urea wash proteins were reduced with 100 mM DTT in 8 M Urea Tris/HCl pH=8.0 (60 min, room temperature) and cysteine groups alkylated with 50 mM Iodacetamid in 8 M Urea/100 mM Tris/HCl pH=8.0 (30 min, room temperature in the dark). The resin was equilibrated with 50 mM NH_4_HCO_3_ and resuspended in 30 µl 50 mM NH_4_HCO_3_ containing 2 µg sequencing grade trypsin (Pierce) and digested on-resin over night at 37°C. Trypsin-released peptides were collected and dried in a SpeedVac (Savant SPD1010, Thermo Fisher). The resin was again extensively washed (as described above) and resuspended in 50 mM NH_4_HCO_3_ containing 0.5 U PNGaseF. After enzymatic release of N-glycopeptides (37°C, overnight) released deglycosylated peptides were collected and dried in a SpeedVac.

### Mass spectrometric analysis

Samples were reconstituted in 0.3% formic acid and peptide concentrations were measured using a NanoDrop spectrometer. 2.5 µg of tryptic peptides and 1 µg of deglycosylated peptides were analyzed in each LC-MS/MS run on an Orbitrap Fusion (Thermo Scientific) coupled to a Dionex Ultimate 3000 (Thermo Scientific) *via* a nanoelectrospray ion source. Samples were loaded on a 2 cm C18 trap column (Acclaim PepMap100, Thermo Scientific) and separated using a 2.5 h (90 min for *S. aureus* stimulation experiments) non-linear gradient (2-80% acetonitrile/0.1% formic acid, flow rate 300 nl/min) on a 50 cm C18 analytical column (75µm i.d., PepMap RSLC, Thermo Scientific). Full MS scans were acquired with resolution 120k and 60k (*S. aureus* samples) at m/z 400 in the Orbitrap analyzer (m/z range 400-1600 for PAMP experiments; 350-1570 for *S. aureus* experiments and patient-derived samples) using an AGC target of 500.000 or 50 ms. MS1 parent ions were fragmented by higher energy collisional dissociation (HCD, 30% collision energy) and fragment ion spectra were recorded in the ion trap in rapid mode (AGC=10.000 or 35 ms; for patient-derived samples: AGC=50% or 250 ms). The following conditions were used: spray voltage of 2.0 kV, heated capillary temperature of 275°C, S-lens RF level of 60%. Samples were run in technical triplicates, patient-derived samples in duplicates.

### Protein identification and quantification

RAW files were searched against the human UniProt database (version 05.2016) with MaxQuant software (44) (version 1.6.17) and Andromeda search engine. The parameters were set as follows: mass tolerances: 10 ppm for parent ions and 0.5 Da for fragment ions; enzyme: trypsin, two missed cleavages allowed; static modification: cysteine carbamidomethylation; variable modification: methionine oxidation, asparagine deamidation (only data of formerly N-glyco-peptide fractions). FDR = 0.01 was set for PSM and protein level. Tryptic peptide fractions and formerly N-glycopeptides were calculated together as fractions of the same sample. Label free quantities (LFQ) were calculated with ratio counts set to 1. The mass spectrometry proteomic data have been deposited to the ProteomeXchange Consortium *via* the PRIDE (45) partner repository with the dataset identifier PXD036036.

### Flow cytometry

For detection of differential expressed cell surface glycoproteins, PBMCs were isolated and cells were left untreated or stimulated with 50 ng/mL LPS, 100 ng/ml Pam3Cys, 10 ng/ml Malp2, or live S. aureus (MOI=5) for 24 h. Cells were harvested and washed in ice-cold 1% bovine serum/PBS supplemented with human Fc-Block (eBiosciences) and labeled with specific fluorescent-dye conjugated antibodies (monocyte marker: CD14-FITC or CD14-APC; CD54-APC, Immunotools; CD35-PE-Vio770, CD44-FITC, CD85j-PE-Vio770, CD208-APC, CD274-APC, anti-SLC1A3-APC, or anti-HLA-Dr-PE-Vio770, all Miltenyi BioTech). After washing, flow cytometry was performed with an Attune Acoustic Focusing Cytometer (Applied Biosystems). For visualization, FCS-files were loaded into FlowJo software version 10. For quantification of fluorescence intensity values, CD14-positive single cells were gated, and the mean fluorescence of APC/Pe-Vio770 or FITC/PE-Vio770 channels was evaluated.

### Data analysis

For statistical analysis of proteomic data, LFQ intensities were loaded into the Perseus (46) software (version 1.6.2.2). After filtering, missing values were replaced by imputation (values from normal distribution, downshift: 2.1, width: 0.2). P-values from 2-sample t-test were adjusted according to Benjamini and Hochberg. Adjusted p-values ≤ 0.05 and fold changes ≥ 1 were considered significant (**Supplementary Table S4**). For analysis of RNA-AmpliSeq-data, RPKM-values were loaded into Perseus software, lg2-transformed and analyzed by 2-sample t-test analysis. P-values were Benjamini-Hochberg adjusted. Results with FC ≥ 1 and p_adj._≤ 0.05 were considered significant. The transmembrane hidden Markov model (HMM) algorithm (HMMTOP server 2.0 (47),) was used to predict putative TMDs in identified glycoproteins. Ingenuity Pathway Analysis (IPA, Qiagen) software was used for enrichment analysis and upstream regulatory factors based on the t-test data calculated in Perseus. Z-scores were calculated based on fold changes and p-values were adjusted according to Benjamini-Hochberg.

## Results

### Tolerance induction in human monocytes induced by PAMPs and live *S. aureus*

**Figure 1:**
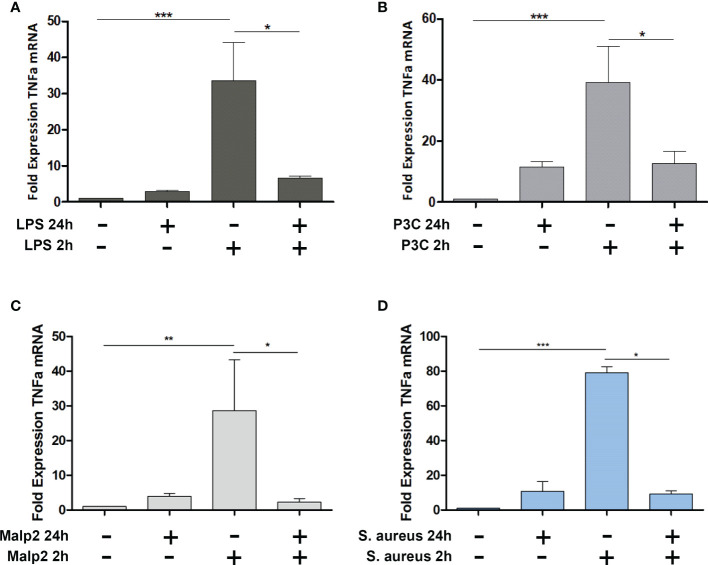
TNF Expression in Malp-2 or *S. aureus* stimulated and restimulated monocytes. **(A–D)** TNF mRNA expression change in monocytes stimulated for 2h, 24h, or prestimulated for 22h and restimulated for 2h with 50 ng/ml LPS **(A)**, 100 ng/ml Pam3Cys **(B)**, 10 ng/ml Malp2 **(C)**, or *S. aureus* **(**MOI=5, **D)** as measured by qPCR (mean ± SD of three independent replicates; statistical analysis was done using 1-way ANOVA and Bonferroni’s multiple comparison post-test; ***p ≤ 0.001, **p ≤ 0.01, *p ≤ 0.05).

To investigate if TLR2 specific ligands and *S. aureus* induce tolerance in peripheral blood monocytes from healthy donors, we stimulated MACS purified monocytes with TLR4 or TLR2 specific ligands LPS, Pam3Cys, or Malp2, respectively and live *S. aureus* bacteria (MOI=5) and monitored TNF mRNA expression after 2h in the pro-inflammatory state, at 24h, and after re-stimulation for an additional 2h time period after 22h of pre-stimulation (**Figure 1**). At a MOI=5 no necrotic or late apoptotic monocytes were detected in flow cytometry after 24h of stimulation with UV-inactivated *S. aureus*, and only a slight increase of early apoptotic cells was detected with live *S. aureus* (**Supplementary Figure S1**). While LPS, Pam3Cys, Malp2 and *S. aureus* led to upregulated TNF transcription at 2h of stimulation, 24h after the initial challenge TNFalpha mRNA level were reduced and comparable with unstimulated control cells. Re-stimulation with a second PAMP challenge or live bacteria for another 2h did not result in upregulated TNF mRNA in the experimental settings, indicating that human monocytes are refractory to second challenges with TLR2 or TLR4-specific ligands and also to Gram-positive *S. aureus* bacteria.

**Figure 2:**
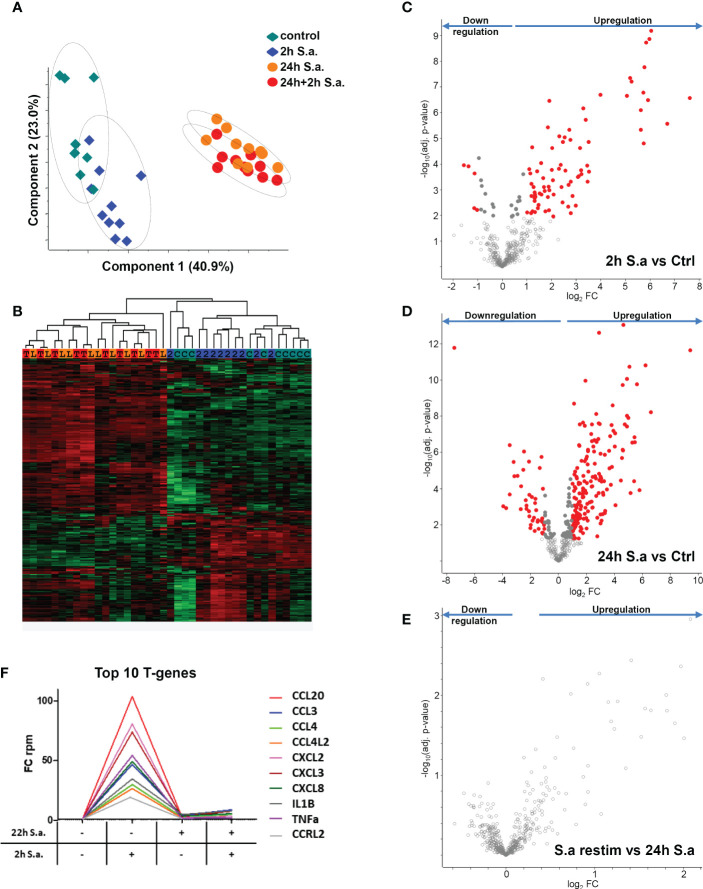
mRNA expression changes in monocytes after *S. aureus* treatments demonstrate tolerance induction revealed by RNA-AmpliSeq. **(A)** Principal component analysis of genes detected in RNA-AmpliSeq. Filled diamonds: unstimulated control samples (grey) and 2h *S. aureus* (blue) stimulated monocytes; filled circles: 22h *S. aureus* (orange) and 22h prestimulated and 2h restimulated (red) monocytes. Note the clustering of individual samples belonging to the different treatment groups (dotted circles). **(B)** Hierarchical clustering of the four treatment groups based on z-scores. The colour coding on top of the graph represents the colouring of treatment groups in A (C=control, 2 = 2h S. aureus, L= 24h *S. aureus*, T= 22h+2h *S. aureus*). **(C–E)** Volcano plots depicting t-test results of gene expression changes in *S. aureus* stimulated monocytes revealed by RNA-AmpliSeq analysis. X-axis: lg_2_ FC, y-axis: -log_10_ p-value **(C)** 2h *S. aureus* stimulated cells compared to untreated controls, **(D)** 24h stimulated cells compared to controls and **(E)** 2h restimulated cells after 22h of prestimulation. Data points of the lower centre area of the plot (grey, open circles) indicate proteins with unchanged or with no significant fold change, whereas data points in the upper left and upper right quadrants indicate proteins (red and grey filled circles) with significant (p ≤ 0.05 after Benjamini-Hochberg adjustment) expression change. Red circles display genes exhibiting fold changes ≥2 and grey circles display significant differentially abundant proteins with FC<2. **(F)** Top10 of tolerized (T-) genes in the AmpliSeq data-set. Depicted is the FC over time according to the stimulation.

To investigate tolerance induction of human monocytes by *S. aureus* infection in more detail, we performed RNA-AmpliSeq sequencing on an Ion Proton semiconductor sequencer with a custom-made primer panel specific for 936 target genes (**Supplementary Table S2**, list of target genes) designed by ThermoScientific. We analyzed mRNA expression levels from ten different donors in the naïve state, after 2h of stimulation with live bacteria (MOI=5), or after 24h to observe long-term transcriptional changes, or after re-stimulation for an additional 2h time period after 22h of pre-stimulation to test for tolerized genes. All samples were sequenced in parallel on a single chip. After filtering 459 sequenced amplicons were evaluable (**Supplementary Table S2**, RPKM values). Principal component analysis (**Figure 2A**) demonstrated that differences in the data set were observable between non-activated and short-term *S. aureus* treated samples (component 2) and more dominantly between long-term and naïve monocytes (component 1). Interestingly, re-stimulated samples were grouped together with 24h stimulation controls revealing only minor differences after *S. aureus* re-stimulation for an additional 2h time period after pre-stimulation. Hierarchical clustering of samples represented as a heat map (**Figure 2B**) demonstrates that expression profiles of monocytes in the naïve state and after 2h of stimulation separated from the 24h treated and re-treated samples, while the short-term stimulations did not result in clear separation of treatment groups due to donor-specific variances in the data-set. After 2h stimulation with live *S. aureus* 102 significant differentially expressed genes (DEGs) were identified in human monocytes and 83 DEGs demonstrated a FC≥2 (**Figure 2C** and **Supplementary Table S3**, FC and p-values of all significant genes). Most DEGs were upregulated after 2h of stimulation (78) and only 5 DEGs were downregulated. After prolonged stimulation for 24h 274 DEGs (217 with FC≥2, **Figure 2D**) were identified in comparison to untreated naïve monocytes. Again, most DEGs showed an upregulation after *S. aureus* stimulation, but 62 DEGs were also significantly downregulated (**Supplementary Table S3**). Restimulation with *S. aureus* for 2h after 22h of pre-stimulation did not result in any detectable DEGs in the comparison to 24h stimulated controls (**Figure 2E**) demonstrating that human monocytes *in vitro* are unresponsive to second challenges with *S. aureus* with regard to gene transcription. According to Foster et al. (8) we next calculated tolerizable and non-tolerizable genes in the data-set of human monocytes stimulated with *S. aureus*. Nine of the TOP10 tolerized genes were pro-inflammatory cytokines and chemokines (CCL20, CCL3, CCL4, CCL4L2, CXCL2, CXCL3, CXCL8, IL1B, TNF), as depicted in **Figure 2F**, and one gene was the transcript for a cell surface receptor, namely C-C chemokine receptor-like 2 (CCRL2). Non-tolerizable genes, with higher FC in re-stimulation experiments were not observed in the data-set. Next, RNA-AmpliSeq results were verified by qPCR analysis for mRNA expression levels of three selected tolerizable genes. The results confirmed that mRNA level of CCL20, IL1B, and CCRL2 were significantly upregulated in the proinflammatory response phase after 2h of stimulation with *S. aureus* and did not rise again after a repeated stimulation 22h after the first *S. aureus* stimulus (**Supplementary Figure S2**).

### Glycoprotein expression changes induced by *S. aureus* stimulation

**Figure 3:**
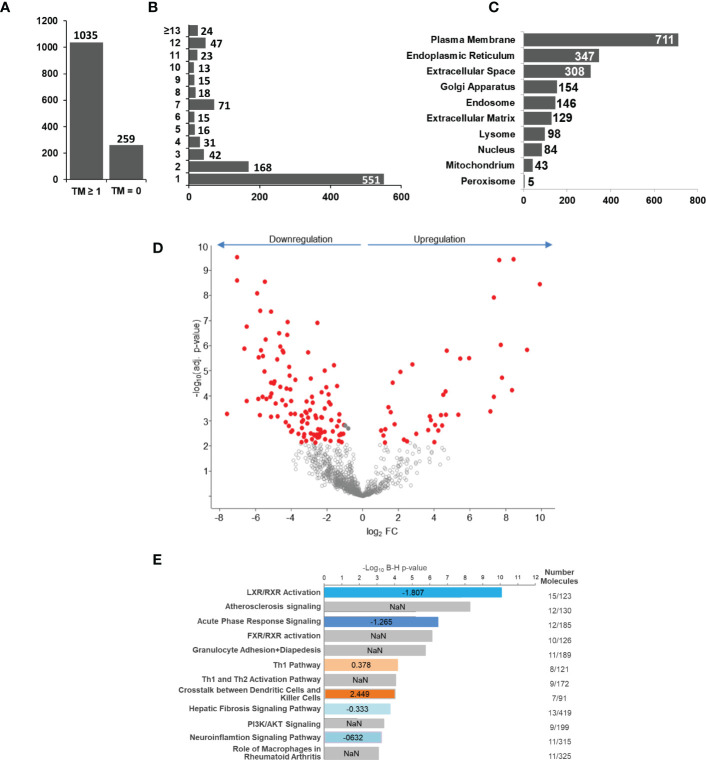
*S. aureus* induced glycoprotein expression changes after 24 h of stimulation. Untreated or *S. aureus* (MOI=5) infected monocytes were lysed and enriched glycoproteins analysed by glycoproteomics. **(A)** Glycoprotein identifications with no or with ≥1 predicted transmembrane domain. **(B)** Distribution of glycoprotein counts with one or more transmembrane domains in the data set. **(C)** Annotated GO categories of identified glycoproteins according to cellular component. **(D)** *S. aureus* induced changes in human monocyte glycoprotein expression profile after 24h of stimulation depicted in a volcano plot (x-axis: lg_2_ fold change (FC) and y-axis: –log10 p-value. Significantly changed glycoproteins after *S. aureus* stimulation in comparison to untreated controls are represented with red filled circles (t-test results, p ≤ 0.05 after Benjamini-Hochberg adjustment. **(E)** Pathway analysis of glycoproteins with significant differential abundance revealed by IPA software. Enriched pathways were sorted according to adjusted p-value. In blue and red enriched pathways with predicted negative or positive activity based on z-score, respectively. Grey bars represent enriched pathways with predicted unaffected or unpredictable (NaN) activity.

Next, we analyzed if human monocyte tolerization mediated by *S. aureus* infection resulted in receptor expression changes detectable on protein level. For this, we examined the glycoprotein expression profiles of naïve and 24h *S. aureus* (MOI=5) stimulated cells from seven healthy donors by glycoproteomics. In the proteomic data set 1.294 proteins were identified with “glycoprotein” annotation in Uniprot database. 259 glycoproteins did not comprise a predicted transmembrane domain and 1.035 comprised at least one (**Figure 3A**). The majority of transmembrane glycoproteins possessed a single transmembrane (551 glycoproteins) domain, others were identified with two or more (**Figure 3B**). Most glycoproteins were annotated with a location at the “plasma membrane” but significant numbers of glycoproteins allocated to intracellular organelles were also identified (**Figure 3C**). Statistical analysis revealed significant changes in glycoprotein expression by human monocytes after *S. aureus* stimulation. Overall 152 glycoproteins demonstrated a significant FC after 24h of stimulation, 38 were significantly higher expressed in *S. aureus* activated cells, and 114 showed a reduced expression (**Figure 3D**; **Supplementary Tables S4** – significant proteins and S5 - all identified proteins). Pathway analysis with Qiagens Ingenuity Pathway analysis software revealed several pathways associated with the observed monocyte glycoprotein changes (**Figure 3E**). Interestingly, “LXR/RXR activation” was highly enriched and calculated with a negative activation z-score. Other enriched pathways with negative z-score were “acute phase response signaling”, “hepatic fibrosis signaling pathway”, and “neuroinflammation signaling pathway” revealing that the tolerant cell state not only has significant impact on transcriptional activity but it also affects protein expression changes associated with a reduction of pro-inflammatory signaling pathways, e.g., the acute phase response (with glycoproteins AHSG, APOH, HP, HPX, ILRAP, IL6R, IL6ST, ORM1, SERPINA1, TNFRSF1A, and TTR demonstrating a significant downregulation and Interleukin-1 receptor antagonist protein/IL1RN a strong upregulation). In contrast, “Th1 pathway” (CD40, CD80, CD274, HLA-DRA, ICAM1 upregulated and IFNGR1 and IL6R downregulated), and “crosstalk between dendritic cells and killer cells” (CCR7, CD40, CD80, CD83, HLA-DRA, IL3RA, and PVRL2 upregulated) were enriched in the data set with a positive activation z-score, indicating receptor expression changes for the activation of pathways involved in adaptive immunity regulation.

### Glycoprotein expression changes induced by TLR2 and TLR4 agonists

**Figure 4:**
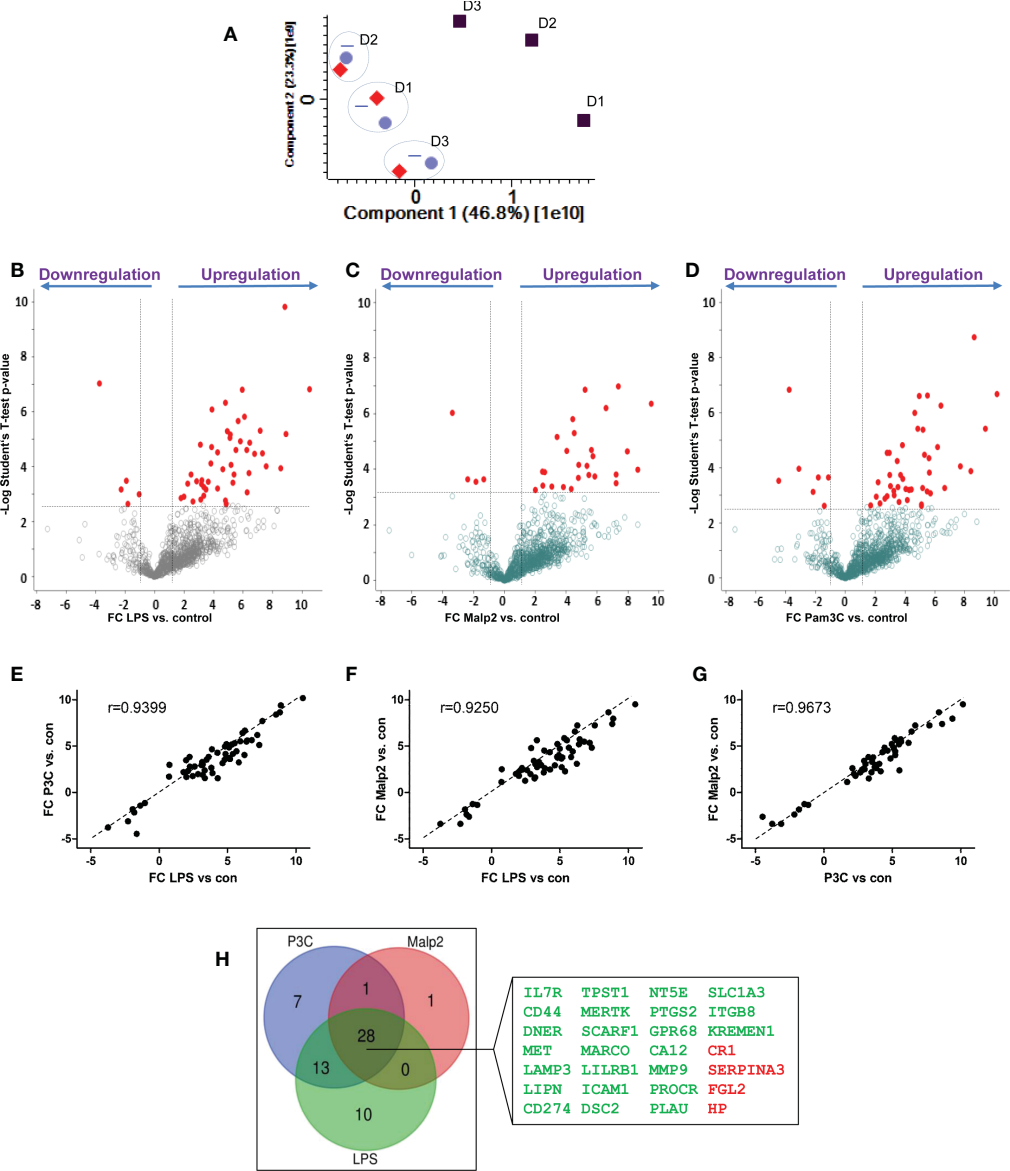
Glycoprotein expression changes after PAMP stimulation in human monocytes. Monocytes of three different healthy donors (D1-D3) were stimulated with LPS (50 ng/ml), Pam3Cys (100 ng/ml), or Malp-2 (10 ng/ml) or left untreated. Glycoproteins were enriched and analysed by LC-MS/MS. **(A)** Principal component analysis, plotted are the two first dimensions. Untreated monocytes (grey squares) separate in the first dimension from LPS (red diamonds), Pam3Cys (blue triangles) and Malp-2 (blue circles). Note the donor-specific clustering of stimulated monocytes (dotted circles). **(B–D)** T-test results depicted in volcano plots with differentially expressed glycoproteins after treatment with LPS **(B)**, Pam3Cys **(C)** or Malp-2 **(D)** highlighted. Glycoproteins with significant expression change (FC≥2, p ≤ 0.05 after Benjamini-Hochberg adjustment) are depicted with red circles. **(E–G)** Linear regression analysis and Pearson correlation coefficient of FC from differentially expressed glycoproteins in the three contrasts, **(E)** LPS vs. Pam3Cys (P3C), **(F)** LPS vs. Malp-2, **(G)** Pam3Cys vs. Malp-2. **(H)** Venn diagram displaying the overlap of differentially expressed glycoproteins in monocytes stimulated with LPS, P3Cys and Malp-2. 28 glycoproteins with a significant up- or downregulation in all three contrasts are highlighted in the blow-up (gene names are given, in green: upregulated glycoproteins, in red downregulated proteins).

Next, we compared the receptor expression profiles of human monocytes tolerized by TLR4 agonist LPS, TLR1/TLR2 agonist Pam3Cys, or TLR2/TLR6 agonist Malp2 to identify a common expression signature indicative for human monocytes in the tolerant cell state. Triplicates of untreated monocytes and LPS (50 ng/ml), Pam3Cys (100 ng/ml), or Malp2 (10 ng/ml) stimulated monocytes (24h) were analyzed. At these concentrations all three ligands led to a reduction of TNF mRNA expression in re-stimulation experiments with LPS (**Supplementary Figure S3A**), demonstrating that TLR4, or TLR1/TLR2, or TLR2/TLR6 activation tolerized and cross-tolerized human monocytes to a second LPS stimulus. In contrast, pre-stimulation with NOD-like receptor (NLR) agonists MDP and iE-DAP did not induce the tolerant state and the cells were fully receptive to LPS stimulation as a second challenge. Reduced TNF and also IL6 expression in the tolerant re-stimulated state was confirmed by specific ELISA on protein level. Here, LPS, Pam3Cys and Malp2 pre-stimulated cells produced significantly diminished levels of TNF and IL6 (**Supplementary Figure S3B, C**) when re-stimulated with LPS. In the proteomic data set of untreated and LPS, Pam3Cys and Malp2 tolerized human monocytes from three different donors 1.194 glycoproteins were identified in total. 1086 ± 21 glycoproteins were identified in the naïve state and after 24h of stimulation with LPS, Pam3Cys and Malp2 1105 ± 22, 1111 ± 16, and 1097 ± 24 glycoproteins were identified, respectively. Principal component analysis revealed that glycoprotein expression in monocytes after 24h of TLR agonist stimulation was distinct from the naïve state. Interestingly, the three different stimulations clustered very closely together, while inter-donor specific differences could be observed (**Figure 4A**). Statistical analysis showed that after LPS, Pam3Cys, and Malp2 stimulation 51, 50 and 30 glycoproteins showed significantly different expression levels, respectively (**Figures 4B–D** and **Supplementary Tables S4** – significant proteins and S5 – all proteins). Linear regression model analysis of differentially expressed glycoproteins after stimulation revealed that the different treatments with TLR4, TLR1/TLR2 and TLR2/TLR6 agonists induced similar expression changes of significant up- and downregulated glycoproteins with Pearson correlation coefficients ranging between r = 0.9250 and 0.9673 with the lowest conformity observed in the comparison LPS vs. Malp2 and highest in the comparison between the two TLR2-specific ligands (**Figures 4E–G**). Indeed, overlap analysis of differentially expressed glycoproteins in the tolerant cell state demonstrated that the three different treatments resulted in highly comparable expression changes with a differentially expressed glycoprotein profile of 28 proteins shared between the three different treatments (**Figure 4H**), and of which most proteins were cell surface receptors. 24 of the shared glycoproteins demonstrated an upregulation, e.g. PD1L1/CD274 with the highest FC (**Supplementary Table S4**), and four glycoproteins were downregulated.

### Shared and differential glycoprotein expression changes induced by *S. aureus* and TLR ligands

**Figure 5:**
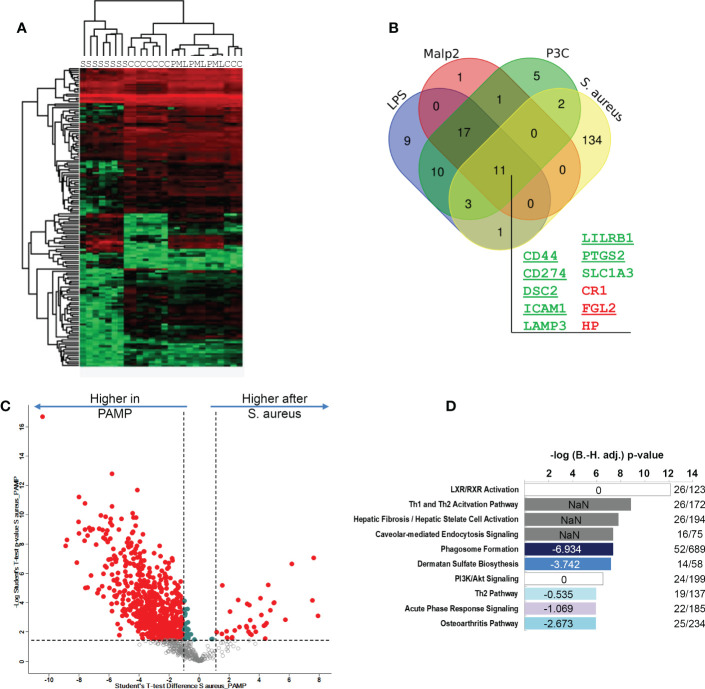
Comparison of *S. aureus* or PAMP evoked glycoprotein expression changes in human monocytes in the tolerant cell state. **(A)** Hierarchical clustering of glycoprotein expression data of TLR ligand stimulated [LPS (L), Pam3Cys (P) and Malp-2 (M)], *S. aureus* (S) stimulated, or untreated **(C)** monocytes. **(B)** Venn diagram depicting the overlap between glycoproteins with significant FC in the four contrasts LPS, Pam3Cys, Malp-2 or *S. aureus* treatment compared to unstimulated controls. 11 glycoproteins collectively up- or downregulated in the four comparisons are highlighted (gene names are given, in green: upregulated glycoproteins, in red: downregulated glycoproteins). **(C)** T-test results depicted as a volcano plot of monocyte glycoprotein expression changes after PAMP (LPS, Pam3Cys and Malp-2 consolidated in one group) and *S. aureus* treatments. X-axis: lg_2_ FC, y-axis: -log_10_ p-value. Glycoproteins with significant differential expression level (FC≥2, p-value ≤ 0.05 after Benjamini-Hochberg adjustment) after stimulation are depicted with red filled circles. **(D)** Pathway analysis of glycoproteins with significant differential abundance revealed by IPA software. Enriched pathways were sorted according to adjusted p-value. In blue enriched pathways with predicted negative activation score based on z-score. White and grey bars represent enriched pathways with predicted unaffected or unpredictable (NaN) activity.

LPS, Pam3Cys and Malp2 induced highly similar changes in glycoprotein profiles expressed by human monocytes in the tolerant state after 24h of activation. To investigate if these protein expression changes were comparable to the changes observed after *S. aureus* treatment of human monocytes, we combined both data-sets and analyzed protein identifications and quantities statistically. Hierarchical clustering revealed that *S. aureus* treated samples separated from TLR agonist treated samples (**Figure 5A**). Next, we compared the significantly changed glycoproteins identified in the three experiments with the different TLR agonists with the glycoproteins significantly changed after *S. aureus* infection. 11 glycoproteins were uniformly up- or downregulated in all 4 comparisons (**Figure 5B**). Eight glycoproteins (CD44 antigen, CD44; Programmed cell death 1 ligand 1, CD274; Desmocollin-2, DSC2; Intercellular adhesion molecule 1, ICAM1; Lysosome-associated glycoprotein 3, LAMP3; Leukocyte immunoglobulin-like receptor subfamily B member 1, LILRB1; Prostaglandin G/H synthase 2, PTGS2; Excitatory amino acid transporter 1, SLC1A3) displayed an increased expression and three glycoproteins (Complement receptor 1, CR1; Fibroleukin, FGL2; Haptoglobin, HP) reduced expression. CD44, CD274, DSC2, ICAM1, LILRB1, PTGS2 and FGL2 were also detected in our quantitative mRNA AmpliSeq analysis with *S. aureus* treated monocytes. In agreement with our glycoproteomic results we found transcripts of all seven proteins either significantly upregulated or, in case of FGL2, downregulated after 24h of stimulation. Furthermore, on transcript level CD274, ICAM1 and PTGS2 were found early upregulated, already detectable at 2h of stimulation. Therefore, this set of 11 proteins represents strong candidates for human monocyte activation markers when the cells entered the tolerant state. qPCR analysis of LILRB1 and CD44 mRNA levels confirmed the results, with significantly elevated expression of LILRB1 mRNA after 24h of stimulation and increased expression of CD44 mRNA at all investigated time points, though significance level was not reached (**Supplementary Figure S4**). Increased monocyte cell surface expression of CD54, CD44 and CD274 was confirmed by flow cytometry after stimulation with LPS, Pam3Cys or *S. aureus* for 24h (**Supplementary Figure S5**). LAMP3 and SLC1A3 on the other hand showed only small amounts of cell surface expression before stimulation and only in case of *S. aureus* treatment, a slight but significant increase in cell surface expression was detected. LILRB1 and, interestingly, also CR1/CD35 cell surface expression was significantly increased after *S. aureus* stimulation but only a tendency of increased cell surface expression was detectable after PAMP stimulation. Next, we were interested in glycoprotein expression differences between TLR agonist stimulated and *S. aureus* stimulated human monocytes. For this, the three highly comparable stimulations with LPS, Pam3Cys and Malp-2, were regarded as one group “PAMP” and compared to the samples stimulated with live *S. aureus* bacteria. 693 glycoproteins displayed significant differential protein abundance with a FC ≥ 2 (**Figure 5C**), and most glycoproteins were expressed at a lesser level after *S. aureus* stimulation when compared to the PAMP stimulations (**Supplementary Table S6**). Pathway analysis with IPA software revealed that the “LXR/RXR activation”,” TH1 and Th2 activation pathway”, “Hepatic fibrosis/hepatic stellate cell activation” and “Caveolar-mediated endocytosis signaling” were most enriched, though without a clear activation trend. “Phagosome formation”, Dermatan sulfate signaling”, “Th2 pathway”, “Acute phase response signaling” and “Osteoarthritis pathway” on the other hand demonstrated a negative activation z-score (-6.934, -3.742, -0.535, -1.069, -2.673, respectively) in the enrichment analysis and were negatively affected by live bacteria treatment compared to TLR ligand stimulations (**Figure 5D**).

### Analysis of patient-derived human monocytes with diagnosed *S. aureus* bacteremia

**Figure 6:**
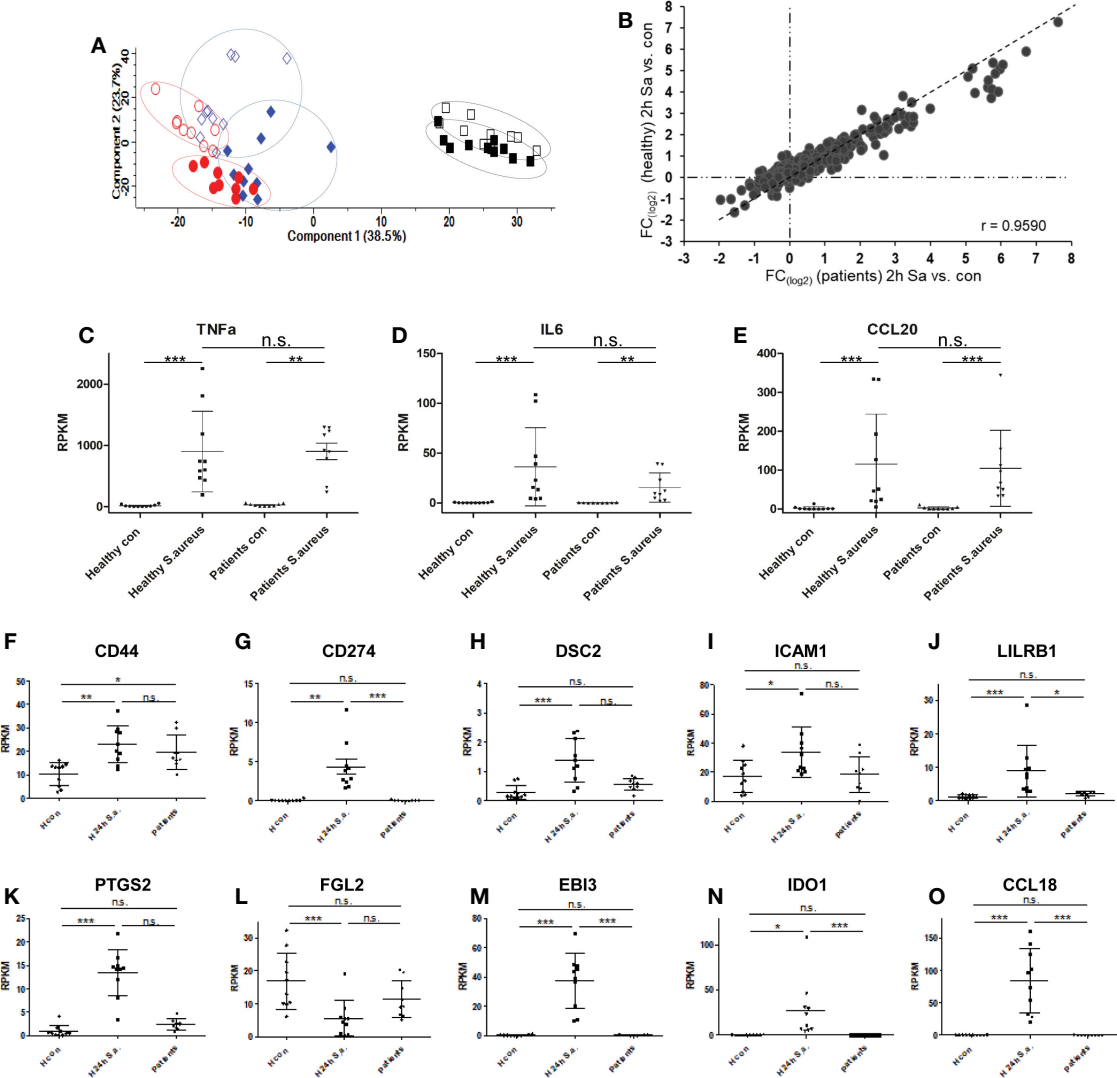
Transcriptional responses of patient-derived monocytes with diagnosed S. aureus bacteraemia after *ex vivo* stimulation with *S. aureus* revealed by RNA-AmpliSeq. **(A)** principal component analysis of RNA-AmpliSeq data from healthy donor monocytes left untreated (blue open diamonds), stimulated with *S. aureus* for 2h (MOI=5, filled blue diamonds), or 24h black open squares, or re-stimulated with *S. aureus* (filled black squares) and patient-derived monocytes directly after purification (red open circles) or after 2h of *S. aureus* (MOI=5) stimulation (filled red circles). **(B)** Scatter plot of mean FC (2h *S. aureus* stimulation vs. Control) from healthy donors (y-axis) and patient-derived monocytes (x-axis). **(C–E)** Individual Expression level (RPKM) of **(C)** TNF, **(D)** Interleukin-6 (IL6) and **(E)** C-C chemokine 20 (CCL20) in monocytes from healthy donors and patients without (con) or after 2h treatment with *S. aureus* (MOI=5). **(F–O)** Individual Expression level (RPKM) of CD44, CD274, DSC2, ICAM1, LILRB1, PTGS2, FGL2, EBI3, IDO1 and CCL18 in monocytes from healthy donors without (H con) or after 24h treatment (H 24h S.a.) with *S. aureus* (MOI=5), or patient-derived monocytes after purification and without stimulation (patients). ***p ≤ 0.001, **p ≤ 0.01, *p ≤ 0.05, n.s. = not significant after 1-way ANOVA and Bonferroni’s Multiple Comparison post-test.

Finally, we aimed to compare our monocyte results from healthy donors with monocytes derived from blood of patients with diagnosed *S. aureus* bacteremia in a small pilot study comprised of 10 patients. Patient characteristics are summarized in **Supplementary Table S1** and changes of CRP plasma concentrations and number of organ dysfunctions over time (day of diagnosis – day of blood sampling – day after blood sampling) are represented in **Supplementary Figure S6A, B**. Two to four days (in nine out of 10 cases) after first positive *S. aureus* blood culture, fresh blood was drawn and monocytes were immediately purified. Changes in CRP levels and recorded organ dysfunctions from 1^st^ blood drawing to analysis indicated that in five cases disease severity was reduced, in four cases unchanged and in one case more severe. One proportion was directly lysed for proteomic analysis of glycoprotein expression, and one proportion was either left untreated or stimulated with *S. aureus* (MOI=5) *ex vivo* for 2h for RNA-AmpliSeq analysis. From nine out of 10 patients RNA amounts sufficient for AmpliSeq analysis was isolated for further analysis. Sequencing of patient-derived monocyte samples was done on the same chip as were the studies with healthy donor samples. Principal component analysis of patient-derived unstimulated, *S. aureus* stimulated samples, and samples from healthy volunteers demonstrated that unstimulated monocyte samples from *S. aureus* bacteremia patients clustered together with the untreated samples of healthy donors (**Figure 6A**). No untreated patient-derived sample clustered together with the *in vitro* activated and 24h tolerized human monocytes from healthy donors on the right side of the PCA plot. In addition, 2h activated patient monocytes showed the same shift in the plot as samples from healthy volunteers. To analyze the pro-inflammatory response of patient monocytes after stimulation with *S. aureus* for 2h in more detail, we then calculated the mean fold changes of DEGs from healthy volunteers and from patients and compared the results (**Figure 6B**). Regarded as groups, the results showed significant correlation (Pearson correlation factor r= 0.9590, p ≤ 0.001), and the data points were equally distributed around the diagonal in the plot. This demonstrates that the pro-inflammatory response of purified monocytes from patients with *S. aureus* bacteremia responded to the same extent during activation *ex vivo* as the monocytes from healthy donors. Next, we examined the transcriptional response detected in RNA-AmpliSeq of three TOP10 tolerizable pro-inflammatory genes, TNF, IL6 and CCLE20 in patient monocytes and healthy volunteers on individual level (**Figures 6C–E**). While there was no transcription of TNF, IL6 and CCL20 detectable in unstimulated cells, after stimulation with *S. aureus* for 2h all three pro-inflammatory gene transcripts were detectable upregulated in healthy donors and patient-derived monocytes. We then analyzed if patient-derived monocytes showed differential transcriptional activity in the expression of genes coding for the glycoprotein marker signature found by our proteomic approach and, additionally, for mRNA expression of the three (EBI3, IDO1 and CCL18) top-most upregulated genes 24h (long-term) after *in vitro* stimulation with *S. aureus*. Corresponding to the expression levels observed in naïve monocytes of healthy volunteers EBI3, IDO1, and CCL18 and CD274 mRNAs were not or at a very low level detected in patient-derived monocytes (**Figures 6M–O, G**). Our proposed glycoprotein marker panel was covered by primers against CD44, DSC2, ICAM1, LILRB1, PTGS2, and FGL2 and for the later five genes no significant mRNA expression change was detected in comparison to healthy control samples (**Figures 6H–L**). In contrast, CD44 mRNA (**Figure 6F**) showed a significant (p ≤ 0.05) upregulation in patient-derived monocytes when compared to untreated healthy donor samples. Elevated CD44 mRNA levels in monocytes derived from patients were subsequently confirmed by qPCR analysis (**Supplementary Figure S7**).

Finally, we examined patient-derived monocytes for glycoprotein expression profiles by proteomics. Due to the varying low numbers of monocytes purified from patient blood, the number of LC-MS/MS identified glycoproteins in patient samples varied markedly and was incommensurable for statistical analysis with our *in vitro* obtained data with monocytes from healthy volunteers. Therefore, we examined the data set for identifications of our marker glycoprotein panel and for the expression of the top-most upregulated genes in the 24h stimulation experiments in individual patient-derived samples on qualitative level. On protein level, Interleukin-27 subunit beta (EBI3), Indoleamine 2, 3-dioxygenase 1 (IDO1), C-C motif chemokine 18 (CCL18), and CD44 antigen (CD44) were not identified in any patient. In activated and tolerized monocytes downregulated proteins Haptoglobin (HP), Complement receptor type 1 (CR1) were identified in all patients and Fibroleukin (FGL2) in 7 out of 10 patients. Intercellular adhesion molecule 1 (ICAM1) and Leukocyte immunoglobulin-like receptor subfamily B member 1 (LILRB1) were identified in nine patients, Desmocollin-2 (DSC2) and Prostaglandin G/H synthase 2 (PTGS2) were only identified in patient No. 10 and Programmed cell death 1 ligand 1 (CD274) was identified in patients No. 1 and 10. Ranking analysis of proteins with an assumed upregulation after *S. aureus* stimulation sorted by abundance in individual samples did not show any significant difference in protein rank for CD274, ICAM1 or LILRB1 in patients when compared to unstimulated controls (data not shown). Taken together, we found no evidence for tolerance induction in monocytes derived from patients with *S. aureus* bacteremia neither on RNA level nor on protein level.

## Discussion

*Staphylococcus aureus* is a major human pathogen colonizing one-third of the human population in nares, throat, or skin but can also cause numerous infections that range from mild to potentially lethal invasive courses (21). The pathogen has developed several mechanisms to evade the human immune system, e.g. *S. aureus* circumvents host humoral defense mechanisms through factors that undermine antibody functions or appropriate B cell development (48), inhibits functional opsonization, avoids killing mechanisms by phagocytes or neutrophils, and it is capable of intracellular survival and biofilm formation (28). In this study, we investigated whether *S. aureus* induces a tolerant state of peripheral human monocytes upon contact with the pathogen and to what extent this feature is associated with changes in cell surface receptor expression. We found that initial contact with vital *S. aureus* provoked a strong inflammatory response in human monocytes with induction of pro-inflammatory cytokines and chemokines as reported by others (49) for Gram-positive bacteria. The pronounced pro-inflammatory gene transcription activity was no longer detectable in subsequent re-stimulation experiments with vital bacteria 22h after the first stimulus. Among other cytokines and chemokines, *S. aureus* and all tested TLR-specific agonists induced significantly increased expression of TNF on mRNA and protein level in the pro-inflammatory phase that was strongly reduced in a subsequent stimulation, indicating that the cells were tolerant to challenges with the same stimulus or exhibited cross-tolerance. The pro-inflammatory cytokine TNF itself was reported to be involved in tolerance and cross-tolerance induction of primary human monocytes and macrophages (50) and might be one critical factor for the observed similarities during tolerance development in our experimental model *in vitro*, since we adjusted all primary stimulations for comparable TNF induction after 2h of stimulation. Abundant inflammatory cytokine production results in excessive inflammation and associated tissue damage and contributes to the pathogenesis of inflammatory disorders. Accordingly, the production of inflammatory signals is tightly regulated by various mechanisms that control the intensity of inflammation and orchestrate the eventual resolution and the return to tissue homeostasis (51). Vital *S. aureus* in the bloodstream and moreover in infected tissues might exploit this modulatory mechanism by creating their own niche through manipulating monocytes and macrophages towards cells with dampened inflammatory potential in their surroundings after an initial contact. In accordance with this, tolerance was recently described in monocytes purified from patients during the acute phase of community acquired pneumonia (9), which can, among other pathogens, be caused by *S. aureus*. Interestingly, patients with community-acquired *S. aureus* bloodstream infections and patients with prolonged bacteremia demonstrate also an increased risk of developing secondary infection foci (52). Tolerance induction in human monocytes might be involved in developing these secondary infection foci due to dampened pro-inflammatory responses after recurrent contact with the pathogen. In our study the examined patients with diagnosed *S. aureus* bacteremia, however, did not show any indication of the tolerant cell state in circulating classical monocytes in blood drawn two to four days after the initial diagnosis. Instead, the examined cells were fully responsive towards *S. aureus* stimulation *ex vivo*, capable of inducing a robust pro-inflammatory response undistinguishable to monocytes from healthy donors. Reasons for this could be the varying severity of infection between the studied patients, the short life-span of circulating monocytes (53), and the long time from first diagnosis to blood sampling for analysis. The only evidence for monocyte activation in patient blood samples with diagnosed *S. aureus* bacteremia that we found was an increased expression level of CD44 antigen. CD44 plays a role in cell-cell interactions, cell adhesion and migration and senses changes in tissue microenvironment, cytokines or growth factors (54). Additionally, CD44 functions to regulate activation of both TLR2 and TLR4 (55, 56) and inhibits of NFκB-dependent pro-inflammatory gene expression in macrophages. As the investigated monocytes from *S. aureus* bacteremia patients were fully responsive towards an *ex vivo S. aureus* stimulation, elevated CD44 mRNA expression and protein cell surface expression can, therefore, only be seen as a monocyte activation marker rather than being a marker of monocyte tolerance. Other markers of the tolerant cell state established by glycoproteomics in this study were detected in amplicon sequencing with either an unaltered expression level when compared to healthy control cells or were not covered by our custom-made primer panel.

Consistent with our glycoproteomic approach with monocytes from healthy donors we found cell surface receptors Programmed cell death 1 ligand-1 (CD274), Desmocollin-2 (DSC2), Intercellular adhesion molecule-1 (ICAM1) and Leukocyte immunoglobulin-like receptor subfamily member-1 (LILRB1) transcriptionally upregulated and Complement receptor type 1 (CR1) downregulated in monocytes from healthy controls after *S. aureus* stimulation for 24h. These cell surface proteins represent strong candidates for surrogate biomarkers of the tolerant cell state of human monocytes in *S. aureus* bacteremia or sepsis. Increased cell surface expression of ICAM1/CD54 and PD1L1/CD274 in the tolerant state was confirmed by flow cytometry after all stimulations, and for LILRB1 after *S. aureus* stimulation. However, for CD35, we detected unaltered or slightly increased cell surface expression after 24h, while the whole cell glycoproteomic approach and also transcriptional analysis by amplicon sequencing demonstrated a downregulation of CR1 expression after 24h of stimulation. In the proteomic data set with TLR-specific ligand stimulations the identified HLA-DR molecules (HLA-DRA, HLA-DRB1, HLA-DRB4, and HLA-DRB5) did not display significant fold changes, and after *S. aureus* stimulation HLA-DRA displayed a slight but significant upregulation on monocytes by glycoproteomics. Cell surface expression analysis of HLA-DR proteins by flow cytometry also demonstrated an upregulation after stimulation of monocytes (**Supplementary Figure S5**), which is in contrast to the reported *in vivo* findings. *In vivo*, decreased HLA-DR expression on monocyte cell surfaces has been demonstrated to be useful as a surrogate marker for detecting monocytes in the refractory state during sepsis (31, 57) where magnitude and persistence of monocyte tolerance is associated with increased risk of mortality and incidence of nosocomial infections (33, 58). The mechanisms responsible for downregulation of HLA-DR cell surface expression *in vivo* are partly known. The imbalance between pro- and anti-inflammatory cytokine responses with increasing anti-inflammatory potential favors diminished HLA-DR expression (59). This condition might not be reflected by the activation of isolated monocytes in cell culture, when the strong pro-inflammatory phase early after stimulation dominates gene expression changes and proinflammatory cytokines are afterwards present at high concentration in cell culture supernatants while anti-inflammatory cytokines like IL-10 are only expressed at later time points. Furthermore, HLA-DR molecules on monocyte cell surfaces are also rapidly regulated, influenced by storage temperature, anticoagulant used for blood drawing, or time until analysis (60), therefore baseline HLA-DR expression in culture might already differ from the *in vivo* situation. Other cell surface receptors reported with reduced expression levels on monocytes during the immunosuppressive phase in sepsis are CX3CR1, CD80, and CD86 (61). CX3CR1 is not an N-glycosylated G-protein-coupled receptor and therefore the protein was not detected in our glycoproteomic approach. With TLR agonists and with *S. aureus* stimulation we found CD86 unchanged on protein level and CD80 displayed an upregulation, though not reaching significance after Malp2 stimulation (**Supplementary Tables S4** and **Table S5**). CD274/PD1L1 on the other hand was found drastically upregulated in monocytes after all treatments. The protein was increased from no or barely detectable protein levels to high expression in all stimulated samples indicating that this protein is a very good candidate as a biomarker of monocyte activation and tolerance. Increased PD1L1 expression by leukocytes in patients with sepsis was reported (34, 62) and a multivariate analysis found that PD1L1 expression level on monocytes is an independent predictor of mortality in sepsis and septic shock (35), when monocytes are in a refractory cell state. As none of the investigated bacteremia patients was diagnosed with septic shock and the majority of patients showed a substantial improvement in the course of the disease until the blood sample for RNA-AmpliSeq investigations was drawn, the lack of CD274 expression in the scrutinized patient’s monocytes is in line with the observed full responsiveness towards *S. aureus* stimulation *ex vivo* and the lack of expression of other tolerance marker. In addition to cell surface receptors, we found two intracellular glycoproteins upregulated and part of the signature of tolerance. LAMP3 is a lysosomal type-1 transmembrane protein predominantly expressed in dendritic cells involved in protein degradation processes and unfolded protein response with implications in microbial infections (63, 64). Flow cytometry of stimulated monocytes revealed that only marginal amounts of LAMP3 reached the cell surface in naïve or tolerized monocytes, similar to results obtained for cell surface protein amounts detected for Excitatory amino acid transporter 1/SLC1A3.PTGS2 is a peripheral membrane protein of the Endoplasmic reticulum with dual enzymatic activity involved in the biosynthesis pathway of prostaglandins with a particular role in the inflammatory response to infections. The enzyme was found upregulated in monocytes by cytokines and TLR ligands (65, 66) but is not detected on cellular surfaces.

After PAMP stimulation with LPS, Pam3Cys and Malp2 we found a surprisingly uniform up- and downregulation of membrane-bound glycoproteins, with the limitation, however, that, to some extent fold changes observed after MALP2 stimulation were less pronounced, not reaching significance level for all differentially expressed proteins detected after LPS or Pam3Cys. The minor differences observed after MALP2 stimulation may relate to the slightly lower number of expressed TLR6 receptors in the investigated untreated monocytes (glycoproteomic data set) in comparison to TLR2 receptors. In contrast, stimulation with *S. aureus* induced more drastic changes in glycoprotein expression mostly differential from the changes induced by the TLR agonists. Whole *S. aureus* bacteria strongly affected the expression of glycoproteins involved in phagosome formation, which is in contrast to reports demonstrating an upregulation of genes involved in phagocytic processes in transcriptome analysis with monocytes from septic patients (11).

In summary, we found that LPS, Pam3cys and Malp2 induced tolerance in human monocytes from healthy donors, which was associated with comparable changes in receptor expression profiles on the cell surface of monocytes. *Ex vivo* stimulation with *S. aureus* also induced tolerance in human monocytes, concomitant with specific and shared glycoprotein expression changes when compared to TLR agonist stimulations. In patients with *S. aureus* bloodstream infections, we did not detect tolerance induction in patient-derived monocytes but CD44 was upregulated on transcriptional level. A possible reason for the missing tolerance induction in the investigated patient-derived monocytes could be that only one patient showed deteriorated disease severity while the other patients showed reduced or unchanged severity at the time of investigation which is an evident limitation of the performed pilot study. To better understand the extent and persistence of tolerance induced by Gram-positive *S. aureus* bloodstream infections for the immunology of the host response, further studies with larger numbers of severe cases, e.g. septic shock patients, or with patients showing increased disease severity after diagnosis, or blood sampling over the complete time course of the disease are needed and may contribute to the development of future strategies for immunomodulatory therapies.

## Data availability statement

The datasets presented in this study can be found in online repositories. The names of the repository/repositories and accession number(s) can be found in the article/**Supplementary Material**.

## Ethics statement

The studies involving human participants were reviewed and approved by Ethics Committee of Jena University Hospital, Jena, Germany. The patients/participants provided their written informed consent to participate in this study.

## Author contributions

MM, and HS designed research; MM, AB, SHO, TK, AS and CB performed research; SHA and MP contributed patient material and information; HS supervised work; TK, CB, and MM analyzed data; and MM and HS wrote the paper. All authors contributed to the article and approved the submitted version.

## Supplementary material

Supplementary Table 2 AmpliSeq primers and AmpliSeq RPKMs.

Supplementary Table 3 AmpliSeq_list of significantly changed genes.

Supplementary Table 4 List of significantly changed glycoproteins.

Supplementary Table 5 List of identified protein groups.

Supplementary Table 6 List of signiificantly changed proteins in contrast PAMP vs. *S. aureus*.
