# Supplementary figures and images for: Staphylococcus aureus induces tolerance in human monocytes accompanied with expression changes of cell surface markers
Source: Front Immunol. 2023 Mar 31;14:1046374. doi: 10.3389/fimmu.2023.1046374 (PMC10104166; doi:10.3389/fimmu.2023.1046374)

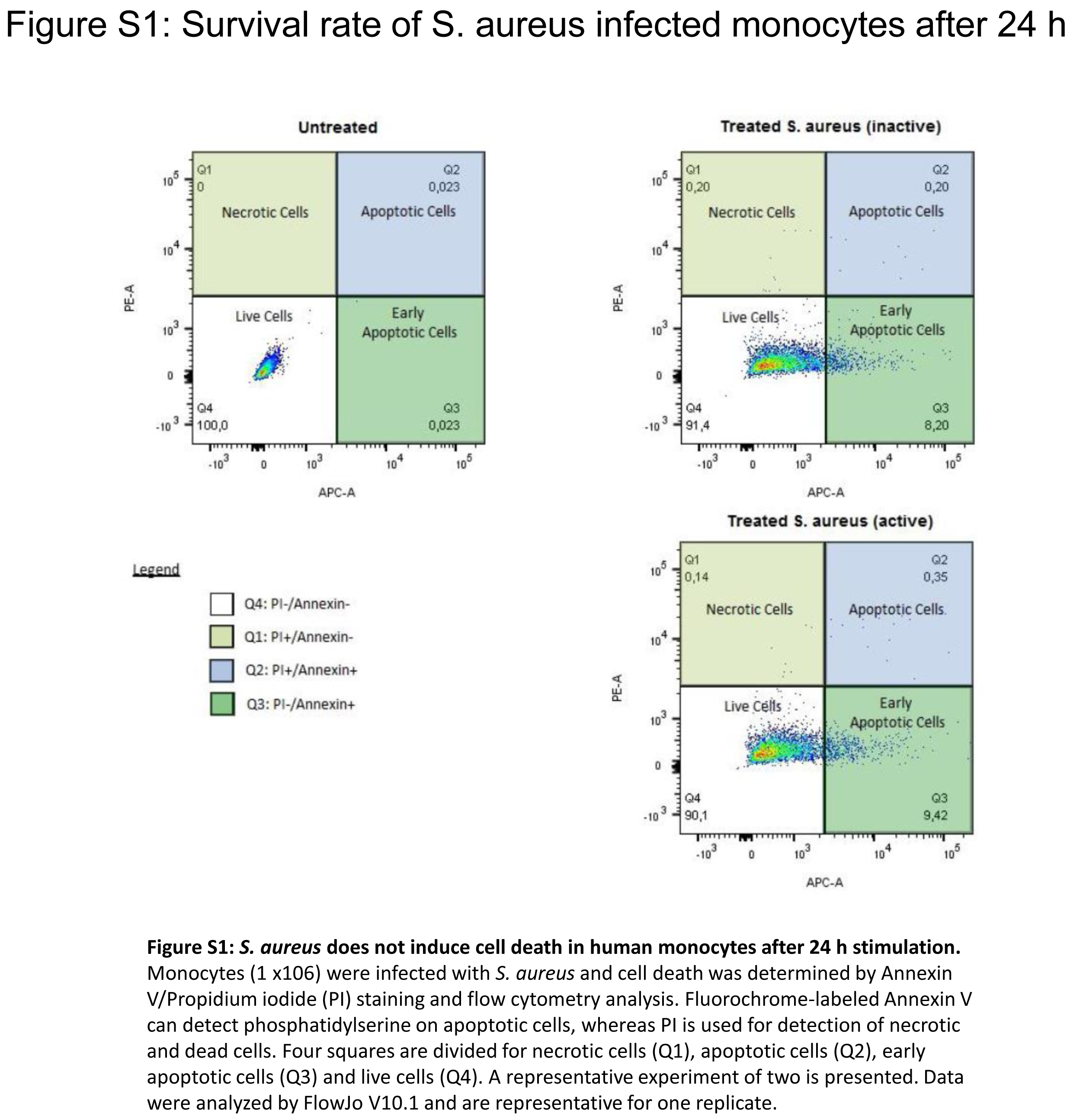

Supplement: Supplementary file 6 [file Image_1.tif]

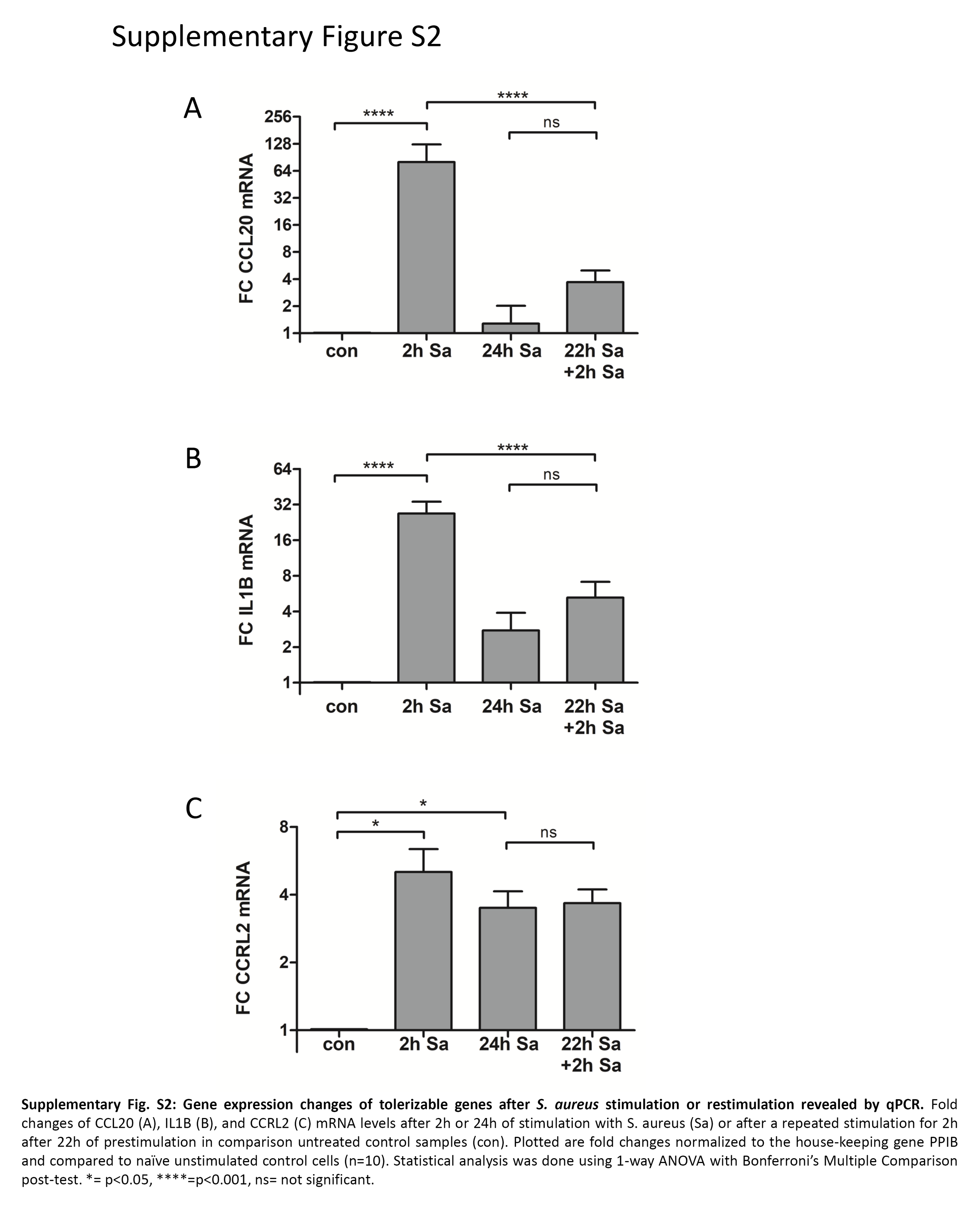

Supplement: Supplementary file 7 [file Image_2.tif]

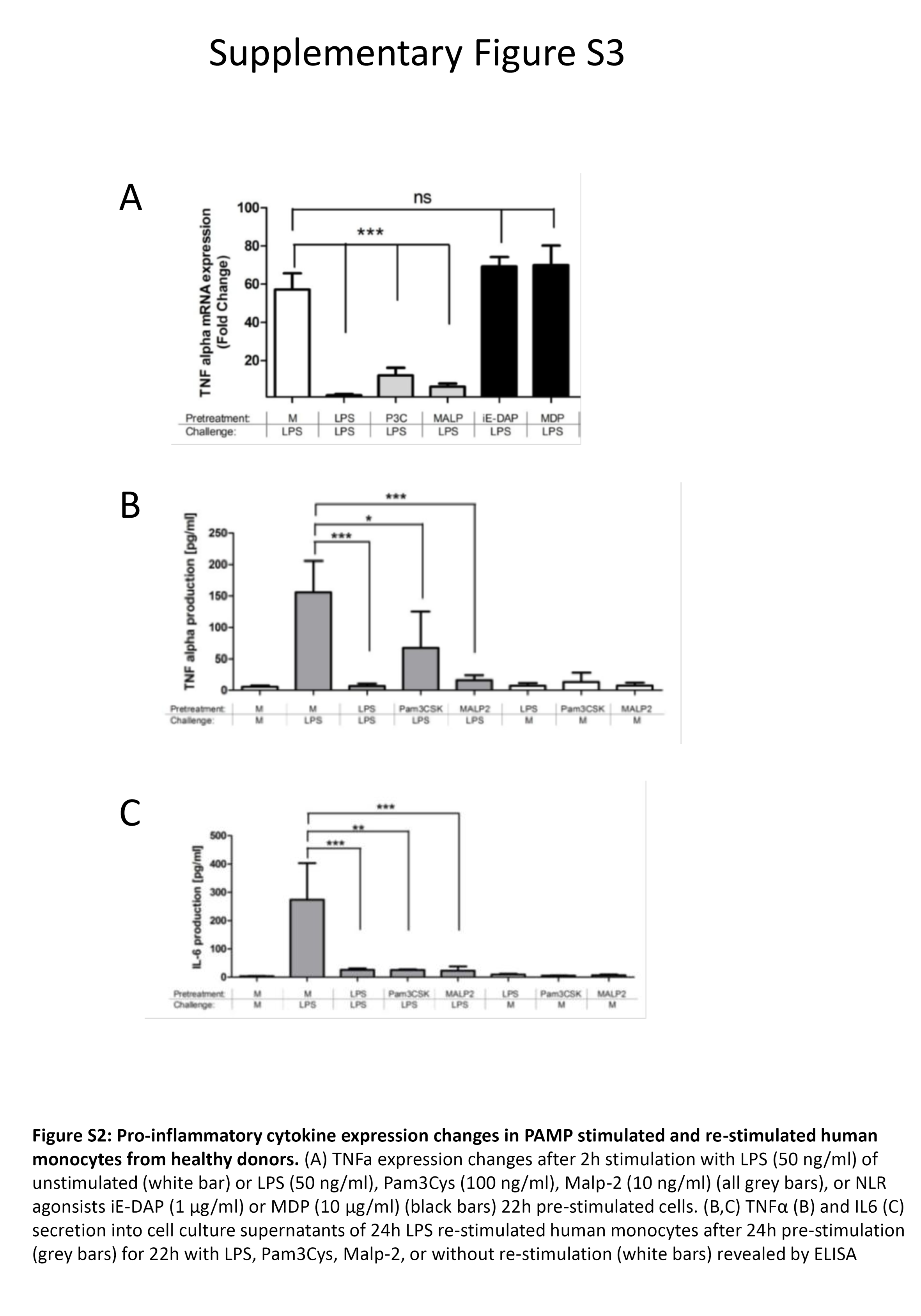

Supplement: Supplementary file 8 [file Image_3.tif]

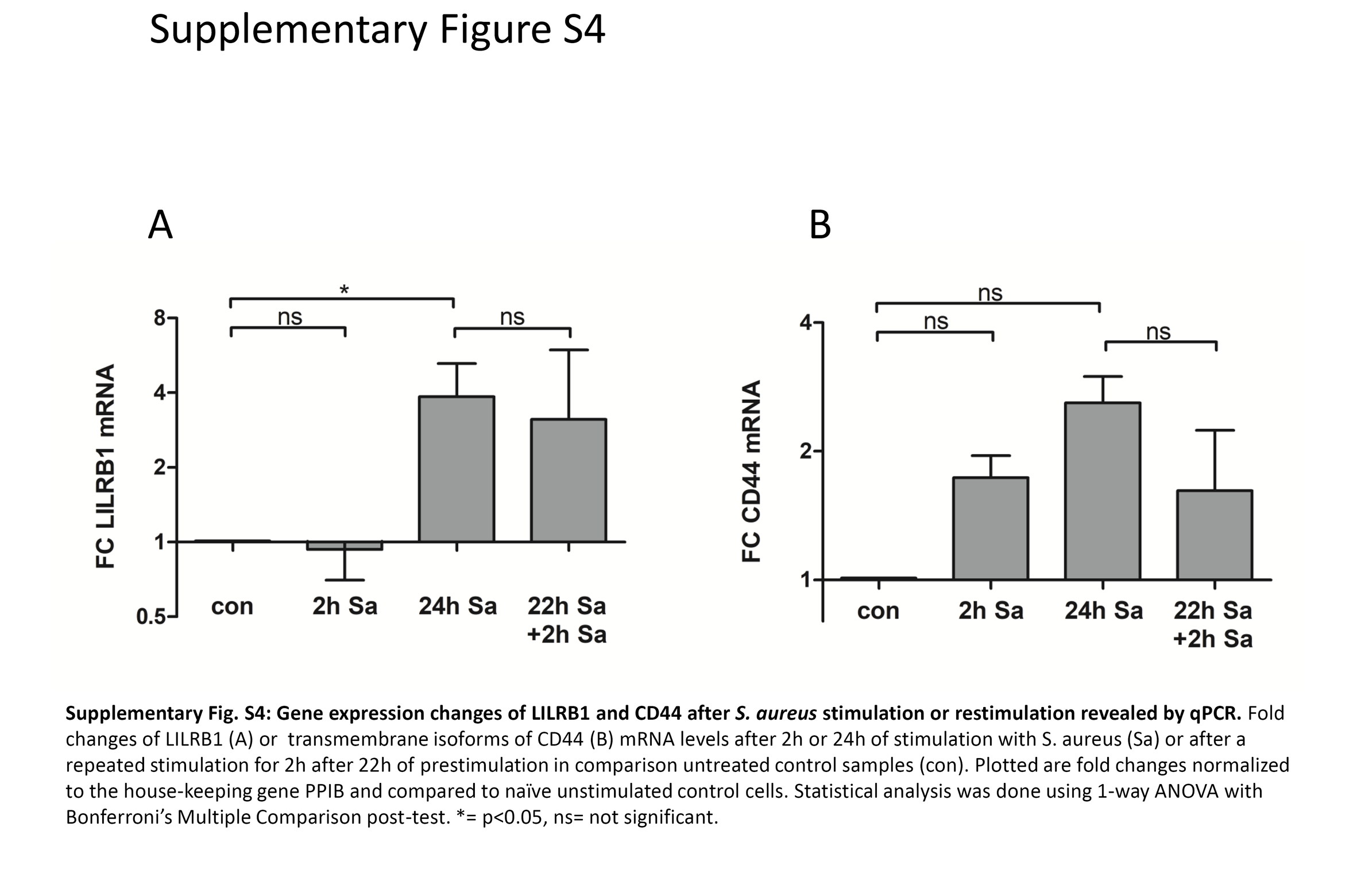

Supplement: Supplementary file 9 [file Image_4.tif]

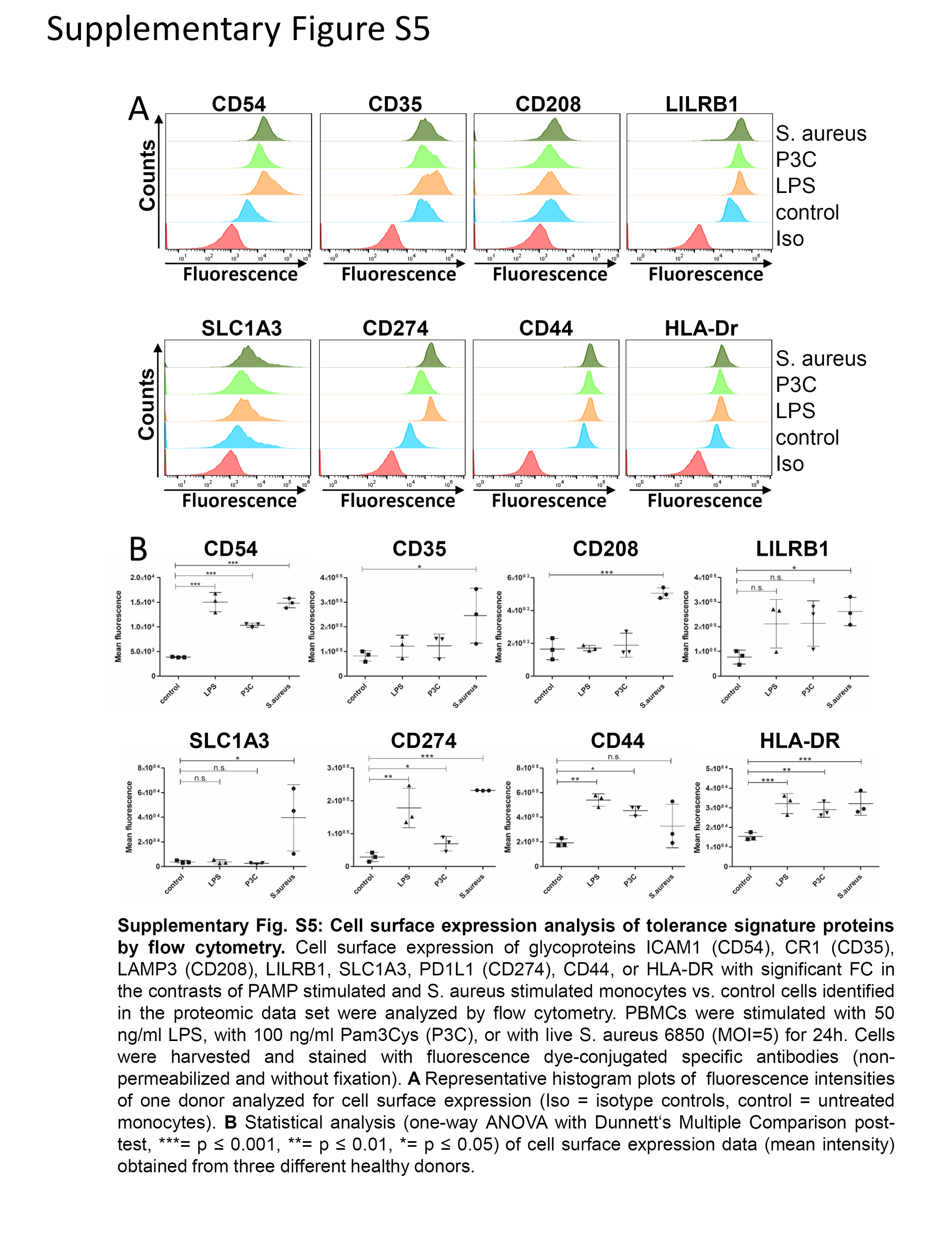

Supplement: Supplementary file 10 [file Image_5.tif]

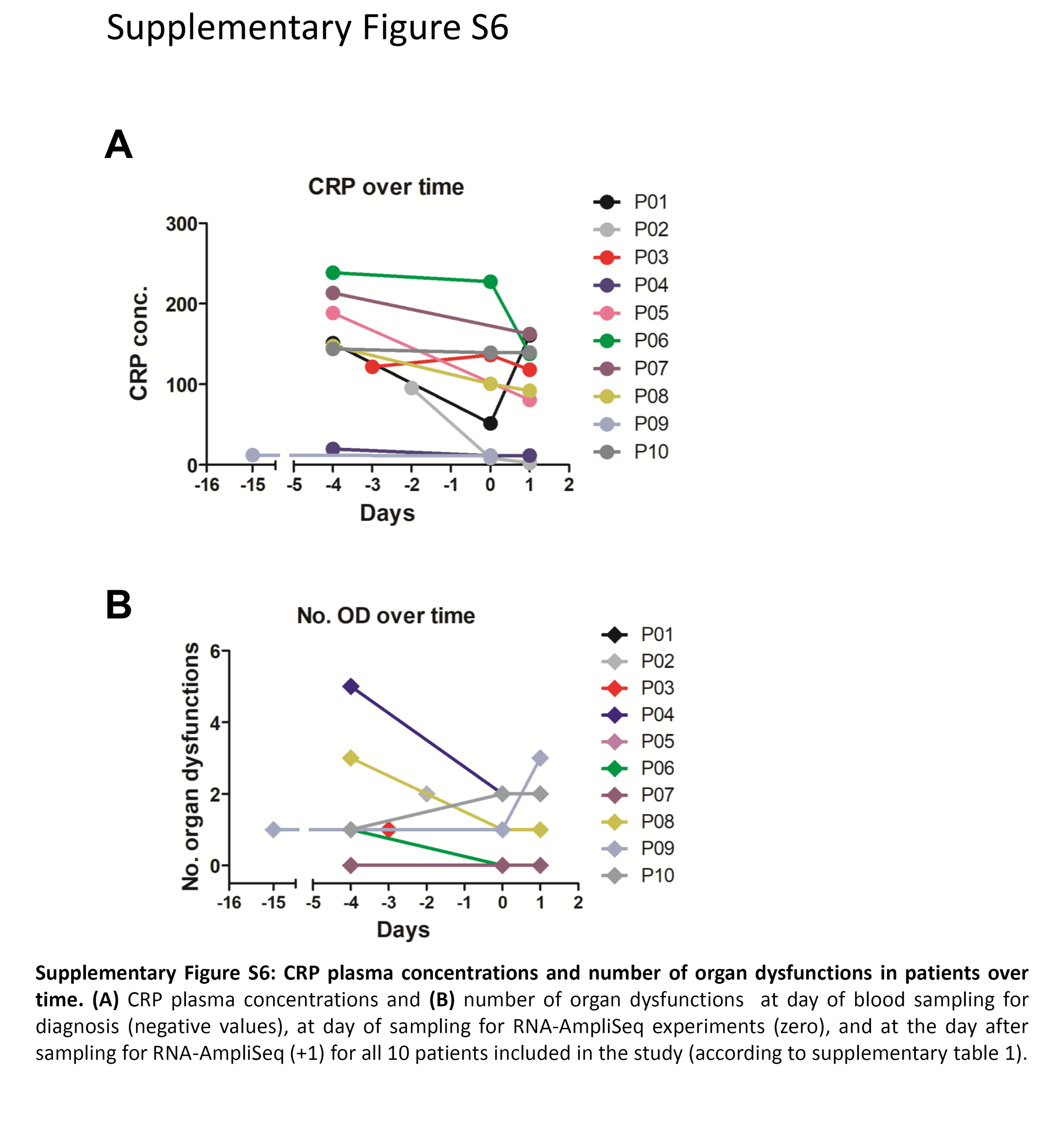

Supplement: Supplementary file 11 [file Image_6.tif]

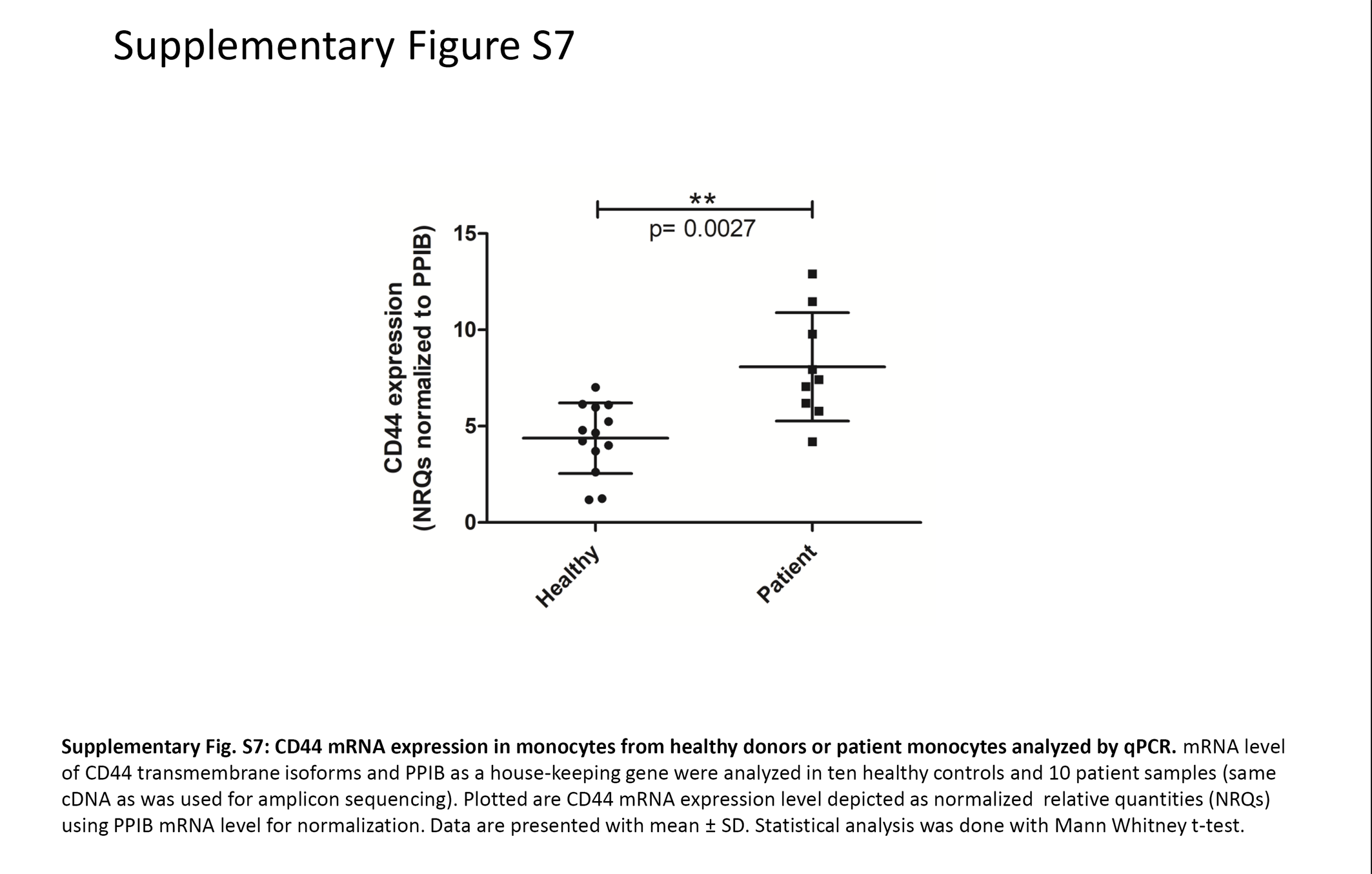

Supplement: Supplementary file 12 [file Image_7.tif]
