# Supplementary material for: Staphylococcus aureus induces tolerance in human monocytes accompanied with expression changes of cell surface markers
Source: Front Immunol. 2023 Mar 31;14:1046374. doi: 10.3389/fimmu.2023.1046374 (PMC10104166; doi:10.3389/fimmu.2023.1046374)
Supplement: Supplementary file 13 [file Table_1.pdf]

Supplementary table 1: Patient characteristics

Patient	Sepsis[§]	Localization of infection	OD[1]	Days after 1st pos. blood culture[$]	CRP (mg/l) day of blood culture	CRP (mg/l) day of analysis	CRP (mg/l) 1 day post analysis	PCT (ng/ml) day of analysis	PCT (ng/ml) high[#]	SOFA[2]	APACHE[3]	Outcome **D**:discharge **M**:deceased	Age	Gender
**P01**	no	Wound healing disorder after coronary bypass	0-0-0	4	151.3	51.3	160.9	0.07	0.07	n.d.	n.d.	**D**; 12 days post analysis	57	m
**P02**	yes	Diverticulum bleeding	2-1-1	2	95.3	8.6	2.4	n.d.	n.d.	n.d.	n.d.	**D**; 12 days post analysis	81	f
**P03**	yes	Catheter infection	1-1-1	3	121.7	136.4	118.2	0.55	0.55	n.d.	n.d.	**D**; 28 days post analysis	79	f
**P04**	yes	Catheter infection	5-2-2	4	19.5	11.4	11.7	n.d.	n.d.	n.d.	n.d.	**D**; 12 days post analysis	87	m
**P05**	yes	Osteomyelitis	2-0-0	4	188.6	n.d.	80.6	n.d.	0.31	n.d.	n.d.	**D**; 18 days post analysis	64	m
**P06**	yes	Superficial open wound infection	1-0-0	4	238.6	227.3	137.9	n.d.	0.57	n.d.	n.d.	**D**;27 days post analysis	75	m
**P07**	yes	Abscess	0-0-0	4	213.2	n.d.	162.3	n.d.	n.d.	n.d.	n.d.	**D**; 25 days post analysis	38	m
**P08**	yes	Lower respiratory tract	3-1-1	4	147.6	100.6	92.1	12.18	16.65	n.d.	26-26-26	**D**; 12 days post analysis	83	m
**P09**	yes	Acute renal failure	1-1-3	15	12.2	11.2	n.d.	n.d.	n.d.	7-n.d.-n.d.	24-n.d.-n.d.	**M**[4]; 1 day post analysis	86	f
**P10**	yes	MSSA Sepsis with acute renal failure	1-2-2	4	143.9	139.1	129.4	n.d.	15.87	8-8-8	31-18-18	**D** (ITS); 1 day post analysis	85	m

[§]: sepsis diagnosed according to sepsis_2 definition;
[$]: time (days) until blood drawn for analysis;
[#]: highest value observed during follow-up after diagnosis
[1]: OD: Number of organ dysfunctions (day of blood culture – day of analysis – 1 day post analysis)
[2]:SOFA-score (day of blood culture – day of analysis – 1 day post analysis)
[3]:APACHE III-score (day of blood culture – day of analysis – 1 day post analysis)
[4]:Death due to renal failure / multiple organ dysfunction
